# GOOGA: A platform to synthesize mapping experiments and identify genomic structural diversity

**DOI:** 10.1371/journal.pcbi.1006949

**Published:** 2019-04-15

**Authors:** Lex E. Flagel, Benjamin K. Blackman, Lila Fishman, Patrick J. Monnahan, Andrea Sweigart, John K. Kelly

**Affiliations:** 1 Bayer Crop Science, Chesterfield, MO, United States of America; 2 Department of Plant and Microbial Biology, University of Minnesota, St. Paul, MN, United States of America; 3 Department of Plant and Microbial Biology, University of California—Berkeley, Berkeley, CA, United States of America; 4 Division of Biological Sciences, University of Montana, Missoula, MT, United States of America; 5 Department of Ecology and Evolutionary Biology, University of Kansas, Lawrence, KS, United States of America; 6 Department of Ecology, Evolution, and Behavior, University of Minnesota, St. Paul, MN, United States of America; 7 Department of Genetics, University of Georgia, Athens, GA, United States of America; Clemson University, UNITED STATES

## Abstract

Understanding genomic structural variation such as inversions and translocations is a key challenge in evolutionary genetics. We develop a novel statistical approach to comparative genetic mapping to detect large-scale structural mutations from low-level sequencing data. The procedure, called **Genome Order Optimization by Genetic Algorithm** (GOOGA), couples a Hidden Markov Model with a Genetic Algorithm to analyze data from genetic mapping populations. We demonstrate the method using both simulated data (calibrated from experiments on *Drosophila melanogaster*) and real data from five distinct crosses within the flowering plant genus *Mimulus*. Application of GOOGA to the *Mimulus* data corrects numerous errors (misplaced sequences) in the *M*. *guttatus* reference genome and confirms or detects eight large inversions polymorphic within the species complex. Finally, we show how this method can be applied in genomic scans to improve the accuracy and resolution of Quantitative Trait Locus (QTL) mapping.

## Author summary

Genome sequences are an essential resource for genetic research in many species. However, most species exhibit considerable variation in genomic organization, making a single reference sequence inadequate. This variation complicates quantitative trait mapping and population genomics. We introduce a new statistical method and computational tools that use linkage information to improve genome assembly and identify structural differences between individuals or populations. We first use the method to correct many assembly errors in the reference genome of *Mimulus guttatus*. Analyzing five crosses from the *M*. *guttatus* species complex, we detect eight large chromosomal inversions and improve the resolution of a trait mapping study. This work illustrates how genetic mapping can be applied to a greater diversity of species to address genetic and evolutionary questions.

## Introduction

Over the last decade, genetic research has been revolutionized by the availability of whole genome sequences for many of the world’s medically, ecologically, and agriculturally important species. It has also become clear that a single reference genome sequence is insufficient for many species. For example, a comparison of two maize accessions found that over 2,500 genes were present in only one of the two genomes [1]. Even in humans, which have much less genetic diversity than maize, structural and gene content polymorphisms are abundant [2]. Differences in gene copy number [3–6], variation in gene order [1, 7, 8] and chromosomal inversions [9–13] are not captured by a single reference genome, nor can they be annotated succinctly in relation to a single reference as is possible for Single Nucleotide Polymorphisms (SNPs). These structural and gene content variants have important phenotypic consequences in many species, highlighting the need for intensive study [14–18].

Recognizing structural variation is important for many of the experimental applications of genomic science. For example, trait-mapping approaches, such as bulked segregant analysis, rely on accumulating signals from adjacent genomic regions (windows) to establish significance. If gene order in the population under study differs from the reference genome, the proximity of polymorphisms will be incorrectly inferred, which in turn, undermines inference of both the location and significance of QTLs [19]. Similar issues can arise in population genomic analyses, such as scans for selection or introgression [20, 21] or of response in “Evolve and Resequence” experiments [22–26]. One solution is to make reference genomes for every divergent accession under study [4]. An alternative approach is to construct ‘pseudo-chromosome’ assemblies to better match structural variation in the focal accessions. Regardless, accounting for structural variation is an important challenge for the continued development of evolutionary genomics.

In this paper, we develop an approach to pseudo-chromosome construction based on comparative genetic mapping. In species with repeat rich genomes, whole genome shotgun sequencing and assembly typically yields many thousands of scaffolds. These scaffolds can be stitched together to form pseudo-chromosomes. There are various techniques for making pseudo-chromosomes, such as following a BAC-tiling path [27], optical mapping using nanochannel arrays [28], or by localizing the scaffolds to markers on a genetic map [29, 30]. Genetic mapping has proved to be effective for initial genome construction and pseudo-chromosome assembly, especially for large genomes [31–33]. We extend this approach using comparative genetic maps from five distinct crosses, allowing us to simultaneously improve the pseudo-chromosome representation of the reference genome and also identify large-scale variation in gene order, including chromosomal inversions and translocations.

Our approach utilizes data from low-coverage sequencing. Restriction site associated DNA-sequencing (RAD-seq) [34, 35] and related reduced-representation methods [36–38] allow cost-effective genotyping of hundreds of recombinant individuals in species with limited genetic markers. While RAD-seq data is often used to create *de novo* markers [39, 40], RAD-seq reads can also be directly mapped to genomic scaffolds. Recombinant genotypes located to genomic scaffolds can then be used to assemble pseudo-chromosomes. Unfortunately, there are substantial challenges in constructing genetic maps from low-coverage sequencing data and in inferring map differences (i.e., the evidence for structural variation). New approaches are needed to address the following methodological questions: What is the optimal means to convert sequence data into markers? How should we accommodate genotyping error in these markers given that the error rate is often high and variable among samples? After locating markers to genomic scaffolds, how do we obtain the optimal order and orientation of scaffolds into pseudo-chromosomes? Finally, how do we determine if putative differences between maps are real?

We develop a statistical procedure called **Genome Order Optimization by Genetic Algorithm** (GOOGA) that detects large structural mutations, such as inversions or translocations, using marker data from multiple genetic mapping populations. Importantly for error-prone low-coverage genotyping, GOOGA propagates genotype uncertainty throughout the model, thus accommodating this source of uncertainty directly into the inference of structural variation. GOOGA couples a Hidden Markov Model (HMM) with a Genetic Algorithm (GA). The HMM yields the likelihood of a given ‘map’ (hereafter used to denote the ordering and orientation of scaffolds along a chromosome) conditional on the genotype data. The GA searches map space by creating new candidate orders which are recurrently fed to the HMM to diagnose their likelihood. Inference of recombination rate parameters and/or tests for differences in gene order are enabled by the fact that all calculations are conducted in the currency of likelihood.

As proof of concept, we first apply GOOGA to mapping data simulated from a known genome sequence. After demonstrating its effectiveness in this context, the pipeline is applied to real RAD-seq data (multiplexed shotgun genotyping (MSG) type [36, 41]) from five different mapping populations, each synthesized from a cross between lines within the *M*. *guttatus* species complex. The complex is a highly diverse clade of inter-fertile North American wildflowers [42–45]. The five mapping populations include both intra- and inter-specific crosses as well as multiple cross types (F_2_s, F_3_s, and recombinant inbred lines (RILs)). Recombinant individuals from each mapping population were scored genome-wide for SNPs and then input to GOOGA. Starting from a rough-draft scaffold order [46], GOOGA produces an optimized order and orientation of genomic scaffolds for each population. The *M*. *guttatus* reference genome derives from an inbred line used as a parents in two of our five crosses, which allows us to correct many errors (misplaced scaffolds) in the reference genome. Improved estimates for recombination rates indicate the effects of gene density and transposable elements on chromosomal variation in recombination. Comparisons among maps identify eight distinct structural polymorphisms, five of which were suggested by previous mapping studies [12, 13, 47–50]. Finally, we demonstrate GOOGA clarifies the results of a QTL mapping study by correcting errors in the reference genome.

## Methods

### A simulation based on Drosophila

We used the "A4" genome build [51] of *D*. *melanogaster* to serve as the reference in the simulation study (downloaded from https://www.ncbi.nlm.nih.gov/assembly/GCA_002300595.1/). This is a high quality build; likely close to the true genome sequence of the line. To estimate multiplexed shotgun genotyping (MSG) [36, 41] of a mapping population using this genome, we extracted MSG reads from a QTL mapping study in *D*. *melanogaster* [52]. We mapped reads, downloaded from https://datadryad.org/resource/doi:10.5061/dryad.gc182, to the A4 sequence using the methods described below for *Mimulus* MSG reads. The mapping locations of restriction site associated DNA-tags (RAD-tags) were thinned to at most one tag per kb, and then used for subsequent simulation of MSG data in a population of F_2_ individuals. To create the mapping population, we consider a cross between isogenic lines that differ at one SNP per RAD-tag. For simplicity, we focused on chromosome 2 with F_1_ flies heterozygous at each RAD-tag along this chromosome. We synthesize each F_2_ by crossing a male that produces gametes without recombination, to a female that produces gametes based on the recombination frequency map of *D*. *melanogaster* [53]. Given the locations and true genotypes at each RAD-tag, we simulate genomic data by sampling a specified number of reads per individual per tag. After forming the entire F_2_ population, we fractured the reference genome into 31 scaffolds by randomly choosing 30 break-locations across the chromosome. The genomic data were then input to the GOOGA pipeline. The genomic coordinates of RAD-tags, the recombination map, and the code to simulate F_2_ genotype and break the reference genome into scaffolds are provided in S1 Data.

### The GOOGA pipeline

**Fig 1:**
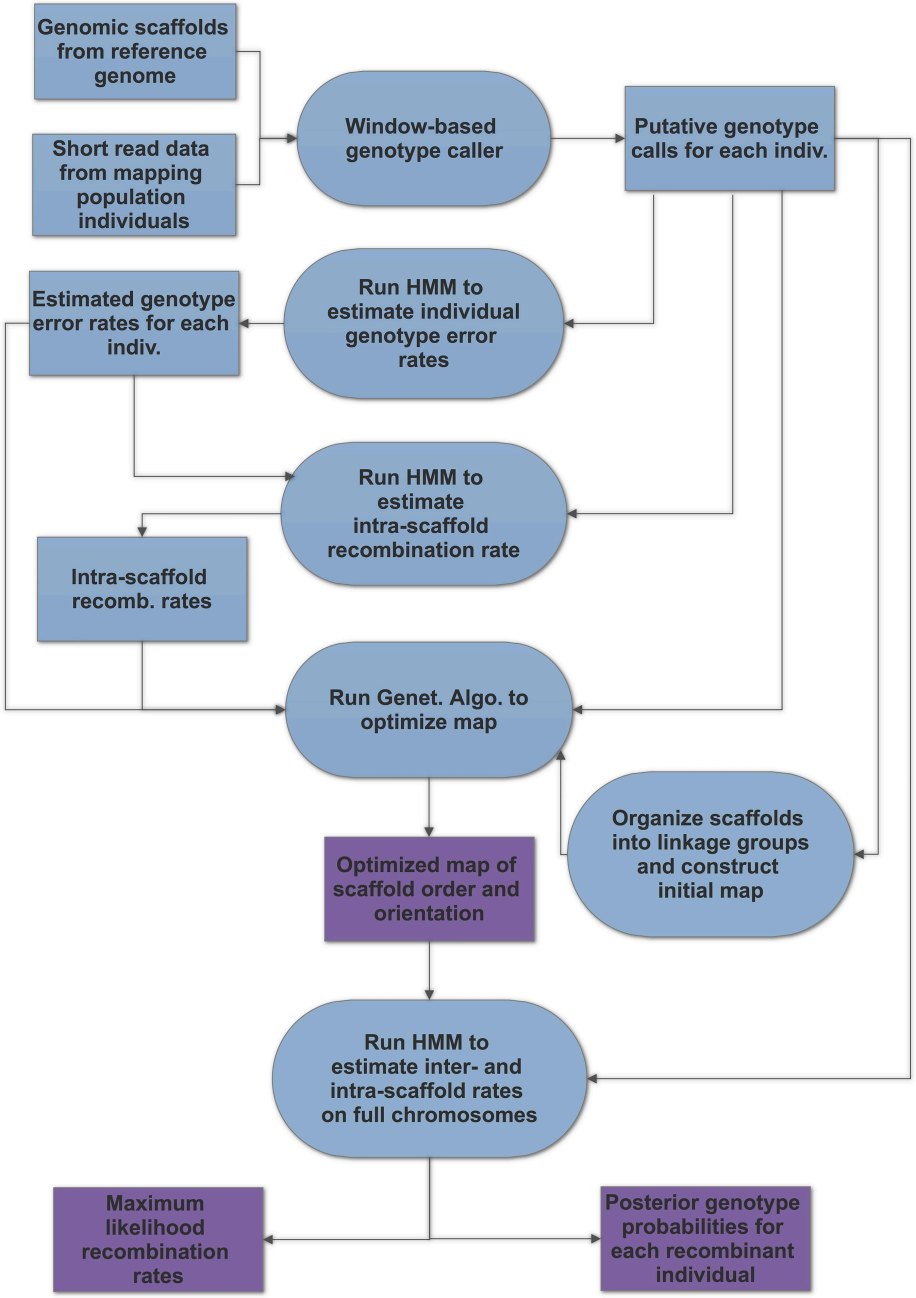
The GOOGA pipeline is illustrated with inputs and outputs (rectangles) and programs (rounded shapes) at each stage. Purple denotes main outputs.

Fig 1 is an overview of the data analysis pipeline.

The first programs take short-read data from recombinant individuals within the mapping population and make “putative” genotype calls. From the parental genomes and offspring of each mapping population we identified SNPs with GATK [54]. We retained only diagnostic SNPs, where bases could be unambiguously assigned to one of the parents (A or B). Window based calls are based on the aggregate of sequence data from all diagnostic SNP sites located between a lower and upper coordinate (all reads that map between these coordinates). The size of windows is a user defined variable chosen based on the density of SNPs and the average number of reads per SNP per individual. The count of reads matching each parent (A or B) is the basis for making putative calls although thresholds for these decisions are also user defined. In our application to both simulated *Drosophila* data and real *Mimulus* data, we delineated markers as windows 100 kb in length within each scaffold. The last marker on each scaffold included all remaining sequence beyond the last complete 100 kb segment. Given the number of reads scored as A and B (across SNPs) within a window, the individual was called AA if the fraction of reads matching parent A exceeded 95%. They were called BB if the fraction was less than 5%, and AB if the fraction was between 25% and 75%. We default to ignorance (NN = No Call) if none of these conditions are met or if the number of reads within the window for that individual is less than a minimum depth threshold (set to 6 for our applications). Ambiguity can result either from mis-mapping of reads (in which case the read counts are misleading) or if recombination occurs within the marker (in which case the true genotype is a combination of two different genotypes, e.g. AA-AB). Scoring either scenario as NN is suitable for downstream analysis by the HMM–data from neighboring markers will strongly inform inference of the underlying genotype at the NN marker. The detailed Methods for making putative genotype calls in for the *Mimulus* data is reported in S1 Appendix.

We use a window-based approach instead of SNP-specific genotyping because the latter can be highly error prone, especially with low coverage sequencing data and mapping to preliminary (error filled) genome builds. Even after numerous filtering steps (see S1 Appendix), SNP-level calls remain problematic (see S1 Fig for an illustration with data from three *Mimulus* recombinants). Under-calling of heterozygotes is often problematic with MSG data; both alleles appear less frequently than the binomial distribution predicts [55, 56]. Aggregating signal from closely linked SNPs addresses both low coverage and allele dropout in heterozygotes. However, SNP level genotype calling may be preferable if both the reference scaffolds and recombinant sequencing are sufficiently high in quality. The downstream components of the GOOGA pipeline are fully compatible with SNP-level calls in this circumstance.

### The likelihood

**Fig 2:**
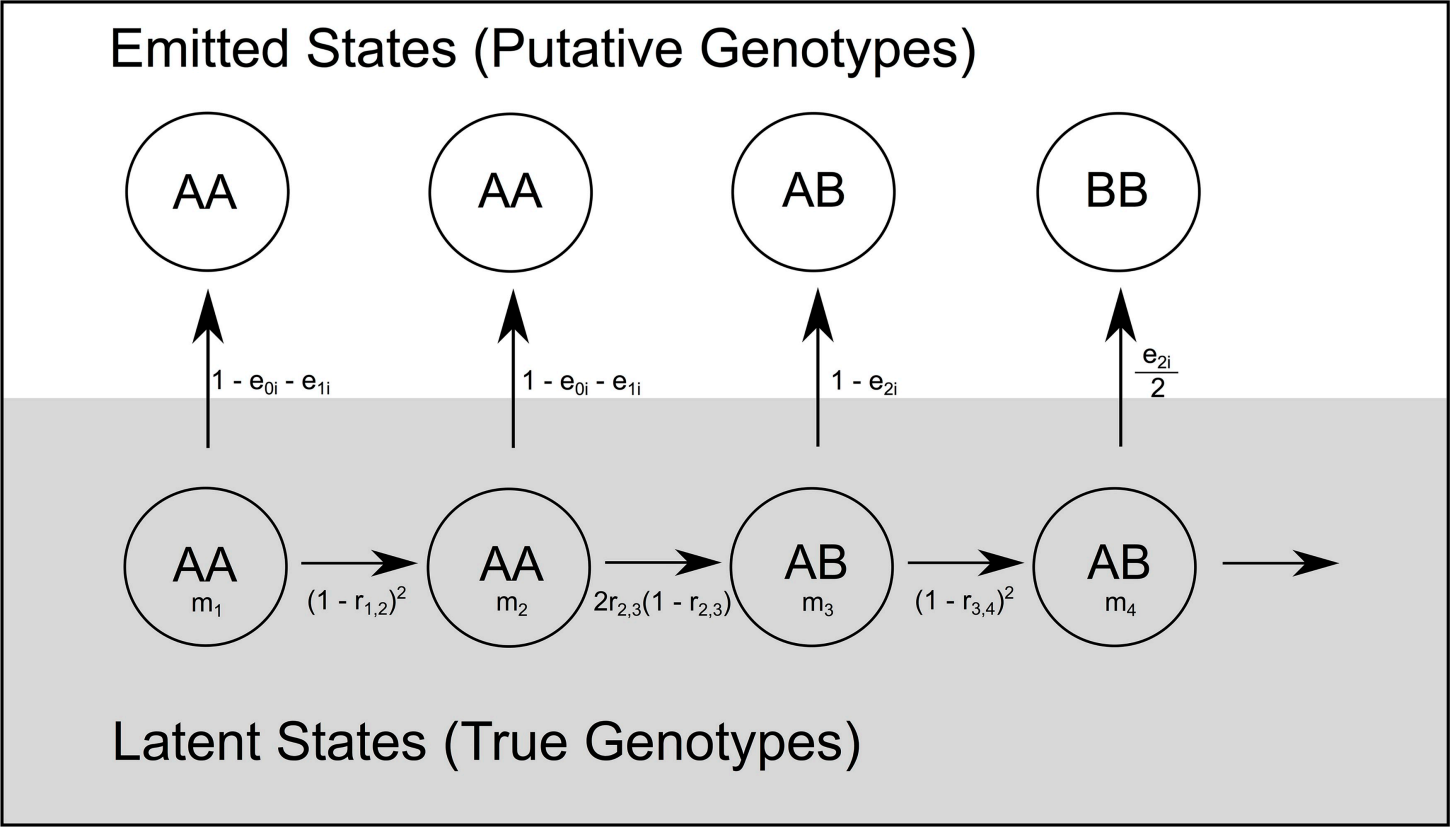
The structure of the Hidden Markov Model is illustrated for F2 individuals. **Transitions between genotype states (AA, AB, or BB) for markers m**_**1**_ **through m**_**4**_ **in the latent state layer are determined by recombination fractions (r) between pairs of markers.** The probabilities of each latent state estimate are then propagated into the emitted state layer with a genotyping error rate term that is specific to each individual (subscript i).

The putative calls are the observed or “emitted” states for the HMM and the hidden states are the true underlying genotypes (AA, AB, or BB) [36, 57, 58]. The structure of the model and transition probabilities depends on the breeding design (e.g. F_2_ or F_3_ or RILs) and on recombination rates. Fig 2 illustrates the HMM for an F_2_ population.

The HMM is non-homogeneous [59] and we report the transitions for F_2_, F_3_, and RIL designs in S2 Appendix. The emission probabilities are determined by individual-specific genotyping error rates (Fig 2). Three distinct error rates are estimated for each individual (i): the probability that a true homozygote yields a putative call to heterozygote (e_0i_) or to the opposite homozygote (e_1i_), and the probability that a heterozygote yields a call to one of the two homozygotes (e_2i_). Regarding the last rate, we assume that errors to either of the alternative homozygotes are equally likely.

The HMM is applied in different ways at four stages of the pipeline (Fig 1). The first objective is to estimate individual-specific genotyping error rates from transitions between genotypes *within* scaffolds. The transition probabilities depend on the true recombination rate per base pair, which is unknown and can vary across the genome. However, to estimate e_0i_, e_1i_, and e_2i_, we fit a simple ‘homogeneous’ model assuming that recombination rate is proportional to physical distance between markers within scaffolds. Based on data from prior *Mimulus* studies [47, 50], we set rates to the genomic average of 5.0 cM/Mb for F_2_ populations and 10.0 cM/Mb for F_3_ and RIL populations. For the simulation study, we use the specified (known) recombination rates. The likelihood of data from each scaffold of an individual plant is then a function of e_0i_, e_1i_, and e_2i_, and the likelihood for the entire plant is a product across scaffolds. For each plant we obtain the maximum likelihood estimation (MLE) of e_0i_, e_1i_, and e_2i_ via application of the forward-backward algorithm [60] coupled with the bfgs bounded optimization routine [61] of scipy.optimize (https://docs.scipy.org/doc/scipy/reference/optimize.html). We also used the bfgs optimizer to obtain MLE for recombination rates as described below. The individual-specific error rates (reported as S1 Table) can be used to cull highly error prone individuals from mapping populations (S2 Appendix).

The next step is to obtain preliminary recombination rate estimates between markers within scaffolds (Fig 1). While error rates were estimated by taking a likelihood across all scaffolds within a single individual, we here maximize the likelihood within each individual scaffold (with respect to r values between all adjacent markers) across all individuals within a mapping population. These intra-scaffold rate estimates are used in the GA search (described below), but not in the final maps. In our application to the five *Mimulus* datasets, the intra-scaffold r estimates were nearly always small, consistent with close linkage. However, we noticed one very high rate between two adjacent markers on scaffold 13 of the DUNTIL cross. The same interval exhibits normal (r < 0.01) recombination rates in other crosses. Given evidence for an inversion breakpoint (further evidence below), we split scaffold 13 into 13a and 13b.

The third application of the HMM is within the GA as it searches for the optimal map (ordering and orientation of scaffolds within a chromosome). Here, the log-likelihood is for the entire chromosome of an individual, summed across all individuals in the mapping population. This requires an assignment of scaffolds to linkage groups. In the absence of other information, this can be obtained by applying a standard map making program such as R/QTL [57] to the putative genotype calls. We wrote a simple end matching program (make.ends.meet.py) to accomplish this task for the simulation study. This program, and all others used in the pipeline, are available at (https://github.com/flag0010/GOOGA).

For a chromosome, there are L– 1 + K recombination rates to be estimated, where L is the number of scaffolds on the chromosome and K is the number of intra-scaffold rates within these scaffolds. The likelihood of a particular map is determined mainly by the data at the “joins” (where two scaffolds meet). The genetic algorithm searches map space with intra-scaffold rates held at their estimates from the second application of the HMM (Fig 1). Evaluation is based on the likelihood of the map after optimizing the inter-scaffold rates. However, we apply the full model, with all intra- and inter-scaffold recombination rates re-estimated, to our final map for each chromosome of each mapping population. In both cases, we obtain the maximum likelihood via application of the forward-backward algorithm.

### The genetic algorithm

GOOGA maximizes the likelihood of the HMM using a genetic algorithm (GA) applied to each chromosome of each mapping population. A GA is an algorithmic optimization scheme inspired by sexual reproduction and natural selection [62]. Below we use terms such as “individual”, “mutation”, “recombination”, and “fitness” as they are frequently used in the GA literature; not to the biological processes. To build the GA, we first coded unique scaffold orders, including scaffold orientations (i.e. forward strand vs. reverse complement), to make an "individual." Each individual represents a candidate solution among a population of size *N* competing in a given generation. The likelihood of the map provides the fitness of individuals. To preserve the best scaffold orders, we used a strategy called elitism (*E*), which allows a predetermined number (*E* = 4 in our case) of the best individuals (i.e., highest likelihood scaffold orders) to go on to the next generation unchanged. To fill the remaining *N-E* spots in the next generation, we applied rank-based selection to select pairs of individuals to “mutate” and “recombine” into new individuals for the next generation. The details of the mating and mutation scheme, and well as the strategy for optimizing computation, are reported in S3 Appendix. After extracting the optimal map from the GA, we run the HMM with both inter- and intra-scaffold rates as free parameters. This yields the final MLE for recombination rates across each chromosomes and also the posterior probabilities of genotypes for each individual in the mapping population.

### Comparing recombination rates to genomic features

We extracted MLE recombination rates from each *Mimulus* mapping population to compare to DNA level features such as amount of coding DNA, number of transposable elements, and the presence of centromeric DNA. For these analyses, we defined a 200 kb interval around each 100 kb-long marker, starting at the midpoint of the preceding marker and ending at the mid-point of the next marker. The analysis is defined at this scale to absorb recombination events that occur mid-marker. Consider the first three markers on a scaffold defined on the position ranges 0–100 kb, 100 kb-200 kb, and 200 kb-300 kb, respectively. We related the sequence interval from 50 kb-250 kb to the sum of the two rates (*r*_1,2_ + *r*_2,3_ of Fig 2). This analysis neglected very small scaffolds, the sequence at the ends of longer scaffolds, and the estimated rates between scaffolds (the amount and the features of the interceding DNA are unknown). In this analysis, we also excluded regions where recombination is suppressed due to inversions (S2 Table). We obtained a single rate for each interval by first standardizing map specific rates by the total length of each map and then calculated a weighted mean across populations. The weight given to estimates from each cross is proportional to the reciprocal of the genome-wide recombination rate variance: IMPR = 1.0, IMSWC = 0.899, IMNAS = 0.790, and DUNTIL = 0.677, and IMF3 = 0.380 (see Table 1 for mapping population abbreviations).

**Table 1:** Details about the five *Mimulus* crosses. For each cross we list the parental lines used, their species, and the cross and mapping population type.

Cross Name	Parent 1 genotype	Parent 1 Species	Parent 2 genotype	Parent 2 Species	Cross Type	Mapping Population Type
DUNTIL	DUN10	*M*. *guttatus*	LVR	*M*. *tilingii*	Interspecific	F_2_
IMNAS	IM160/IM767	*M*. *guttatus*	SF5	*M*. *nasutus*	Tri-parental Interspecific	F_2_
IMSWC	IM62	*M*. *guttatus*	SWC	*M*. *guttatus*	Intraspecific	F_2_
IMPR	IM767	*M*. *guttatus*	PR	*M*. *guttatus*	Intraspecific	RIL
IMF3	IM62	*M*. *guttatus*	IM767	*M*. *guttatus*	Intraspecific	F_3_

### QTL mapping in the IMSWC population

We compared quantitative trait locus (QTL) mapping results for the IMSWC cross using either the optimized scaffold order generated by our pipeline or the scaffold order of the *M*. *guttatus* V2 reference genome. For each of the 873 F_2_s used for genetic map construction, genotype posterior probabilities were emitted for each of the 111 markers defined for chromosome 11. This chromosome harbors a major QTL that contributes to variation in the ability to flower under 13/11 hours of light/dark (Kooyers et al. 2018, in prep.). Genotypes were then assigned at each marker for each individual based on the genotype with a posterior probability > 0.95; otherwise, the genotype was called as missing. For each marker order, we calculated recombination frequencies (Haldane map function), imputed genotype probabilities at 1 cM steps (error probability = 0.001), and performed interval mapping using the binary model in R/QTL [57].

## Results

### The efficiency and limitations of GOOGA are demonstrated by simulated data

**Fig 3:**
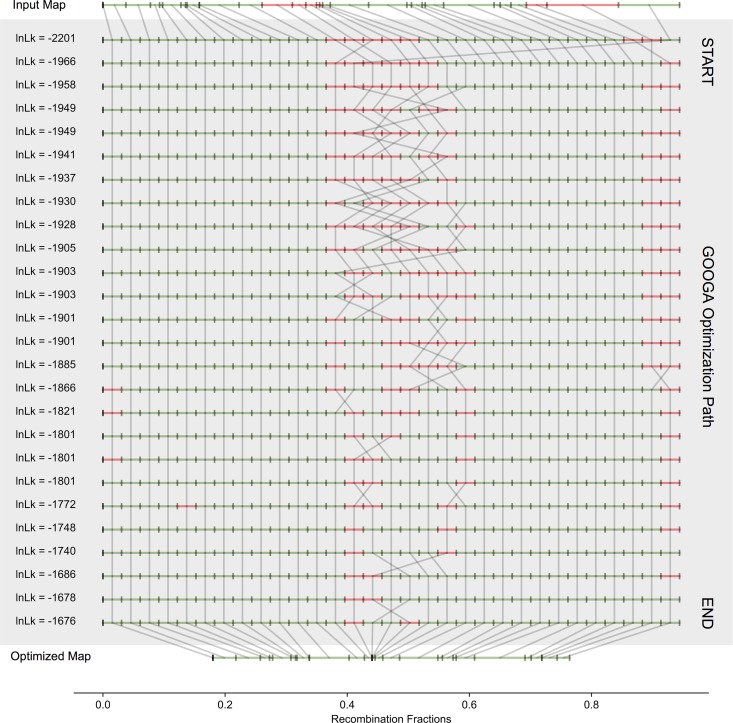
A typical GOOGA run on simulated data for *D*. *melanogaster* Chromosome 2 (200 F2 individuals genotyped for 31 scaffolds). The input map is given on top and the final optimized map at the bottom. The grey shaded region in between are the 26 steps GOOGA took to transform the input order into the final optimized map. The scaffolds in the grey shaded region are all drawn to a uniform length, while the scaffold lengths for input and final maps are drawn to their genetic map length. Green indicates forward orientation, red reverse. Grey lines connect the same scaffold between steps. The log likelihood (lnLk) value for each step is given to the left of the map order.

We simulated data with *Drosophila* chromosome 2, fragmented in different ways into 31 scaffolds, which were then input to the GOOGA pipeline. Fig 3 is a typical result.

The initial map, constructed from putative genotype calls at scaffolds ends, is misassembled in several places, mainly where recombination rates are low. GOOGA converged in 26 steps for this simulation replicate, increasing the log-likelihood (lnLk) by over 500 and reducing the map length by ca. 40%. Genetic map shrinkage occurs because maximum likelihood adjustments of the rate parameters will compensate for bad joins by increasing recombination fraction (r) values. Over the course of a run, bad joins are corrected. Importantly, the final GOOGA order is not completely correct–two small scaffolds near the centromere are inverted. The likelihood of the data under the GOOGA final map and the correct map are exactly equivalent. There are simply no recombinant individuals in the mapping population to resolve the orientation of these scaffolds because it is a low recombination region. In all of simulation replicates, GOOGA converged on the correct map or on a map with an equivalent likelihood to the correct map.

### GOOGA optimization rebuilds the *M*. *guttatus* reference genome

We used GOOGA to optimize chromosome-scale genetic maps for all 14 chromosomes among five *Mimulus* mapping populations (Table 1, S2–S15 Figs, and S3 Table). In each case the current *M*. *guttatus* genome assembly was used as the starting point with 100 kb windows as genetic markers. For each chromosome, GOOGA produced an MLE pseudo-chromosome construction. The overall map lengths for the F_2_ crosses are 1278 cM for the DUNTIL cross, 1523 cM for the IMNAS cross, and 1258 cM for the IMSWC cross. The average map length, 1353 cM, is shorter than previous F_2_ maps generated in *Mimulus* through PCR-based genotyping methods [12, 47, 50]. The map from the IMF3 cross is ≈50% longer than the F_2_ average (2043 cM), as expected given the extra generation of recombination between F_2_ and F_3_ generations. Finally the map from the IMPR cross (1489 cM) is only slightly longer than the F_2_ average. There is additional recombination in the formation of these RILs, but the recombination parameter is specified differently (as related to crossover events) in the RIL HMM (see S2 Appendix).

**Fig 4:**
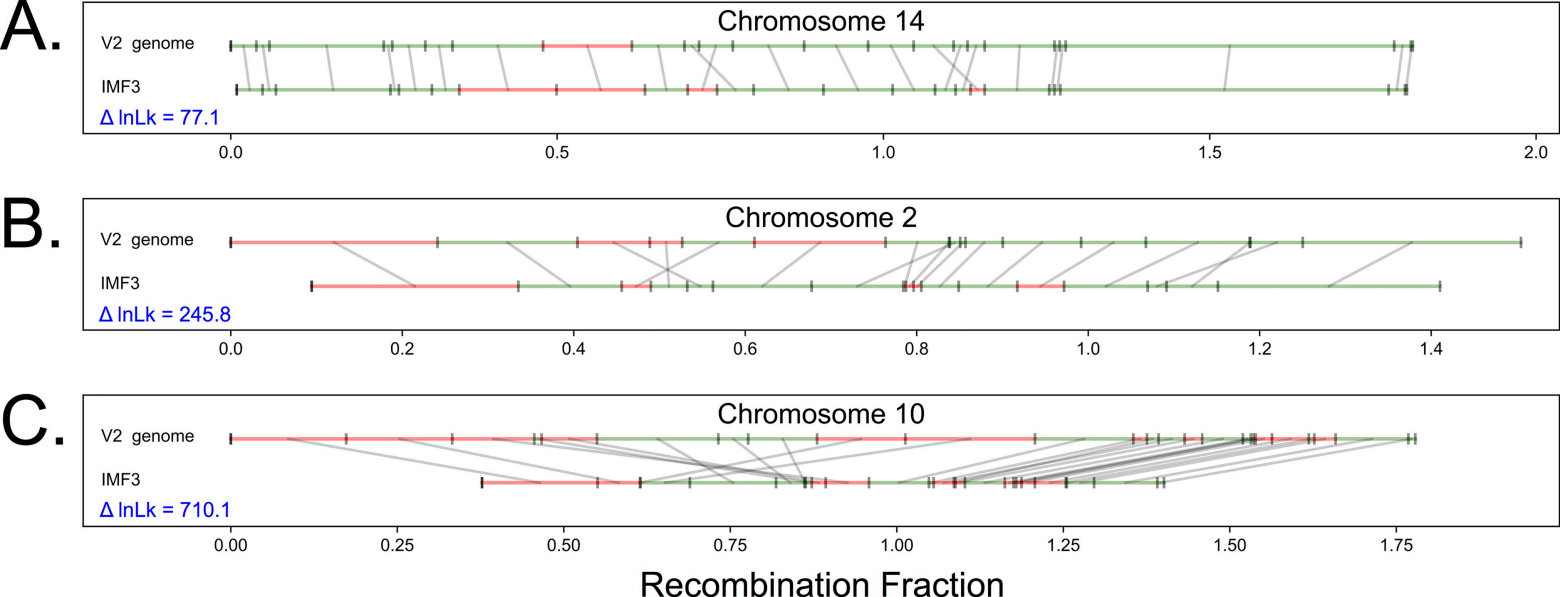
The improvement of the chromosome map between the *M*. *guttatus* V2 reference genome and the GOOGA optimized order for three chromosomes (IMF3 cross). Within each panel, the V2 order is shown above the IMF3 optimized order. Each genomic scaffold is drawn to its genetic map length (in Morgans) and denoted in green if it maps on the forward strand or red for the reverse strand. Grey lines connect the same scaffold in the V2 and optimized order.

Comparison of the five GOOGA optimized maps to the *M*. *guttatus* V2 reference genome order (hereafter V2 map; [46]) indicates that a large number of updates to the reference genome are necessary. Although the five mapping populations differ importantly from each other, changes in scaffold order and orientation from the V2 map are nearly always shared–the five estimated maps differ from the reference in the same way. These regions likely reflect errors in genome assembly given that the reference genome was sequenced from an inbred line (IM62) that is used in two of the crosses that we analyze here (IMF3 and IMSWC). Fig 4 illustrates this point, comparing the maximum likelihood map of the IMF3 data from GOOGA to the V2 map for three chromosomes.

This intra-population cross is likely to be most congruent with the true order of the IM62 reference genome. The difference in log-likelihood (ΔlnLk) provides a measure of improvement in fit of the GOOGA relative to V2. ΔlnLk is computed by fitting the genotype data to both the GOOGA optimized and V2 maps, and then subtracting the former from the latter. In each case, recombination rates are estimated independently, and so ΔlnLk is determined entirely by differences in scaffold order and orientation. The maps for chromosome 14 (Fig 4A) are largely similar. However, differences such as the changes in location and orientation of scaffolds 127, 211, 178, and 140b (inconsistency near the center of Fig 4A, S3 Table), are sufficient for a large improvement in likelihood. The ΔlnLk of 77.1 (S4 Table) corresponds to a likelihood improvement of 10^33^, suggesting this new order fits the segregation data in the IMF3 population far better than the V2 map. Importantly, the updated ordering of scaffolds 127, 211, 178, and 140b is shared by the IMSWC, DUNTIL, and IMPR maps (S14 Fig and S3 Table). The IMNAS map retains the [211,127] ordering of the V2 map, albeit with a flip of 127. However, this inconsistency is not compelling because genome assembly by genetic mapping will fail when there is no recombination to provide signal, and there is no evidence of recombination in this region among the 91 F_2_s genotyped in the IMNAS population.

Chromosome 2 (Fig 4B) is typical of most IMF3 contrasts. Numerous scaffolds are rearranged (e.g. the IMF3 sequence [44a, 212, 249] is inverted in the V2 map) and several are flipped in place (including scaffold 81 that flanks [44a, 212, 249]). There is an increase in likelihood from the V2 map to the GOOGA optimized order (ΔlnLk = 255.8), and the genetic length of chromosome 2 shrinks by ≈15% from V2 to GOOGA. This effect is even more pronounced for chromosome 10 (Fig 4C), where there is a large increase in likelihood (ΔlnLk = 710.1). Among 70 chromosomes (5 crosses x 14 chromosomes), 23 (33%) chromosomes had ΔlnLK improvements greater than 500, while only 5 (7.1%) improved less than 100 (S4 Table). The most significant alterations to the V2 map are in genomic regions harboring inversions, particularly on chromosomes 5, 8, and 10 (Fig 4C, S6 Fig, S9 Fig, and S11 Fig). The V2 map is based partly on genetic mapping data from approximately 70 recombinant inbred lines from a cross between IM62 and DUN10 (J. Willis pers. comm.). DUN10 is a parent in our DUNTIL cross [35] and the aggregate of evidence (see below) suggests that DUN10 has chromosomal inversions (relative to IM62) on each of these chromosomes.

### Comparison of GOOGA maps confirms extensive structural diversity in *Mimulus*

**Fig 5:**
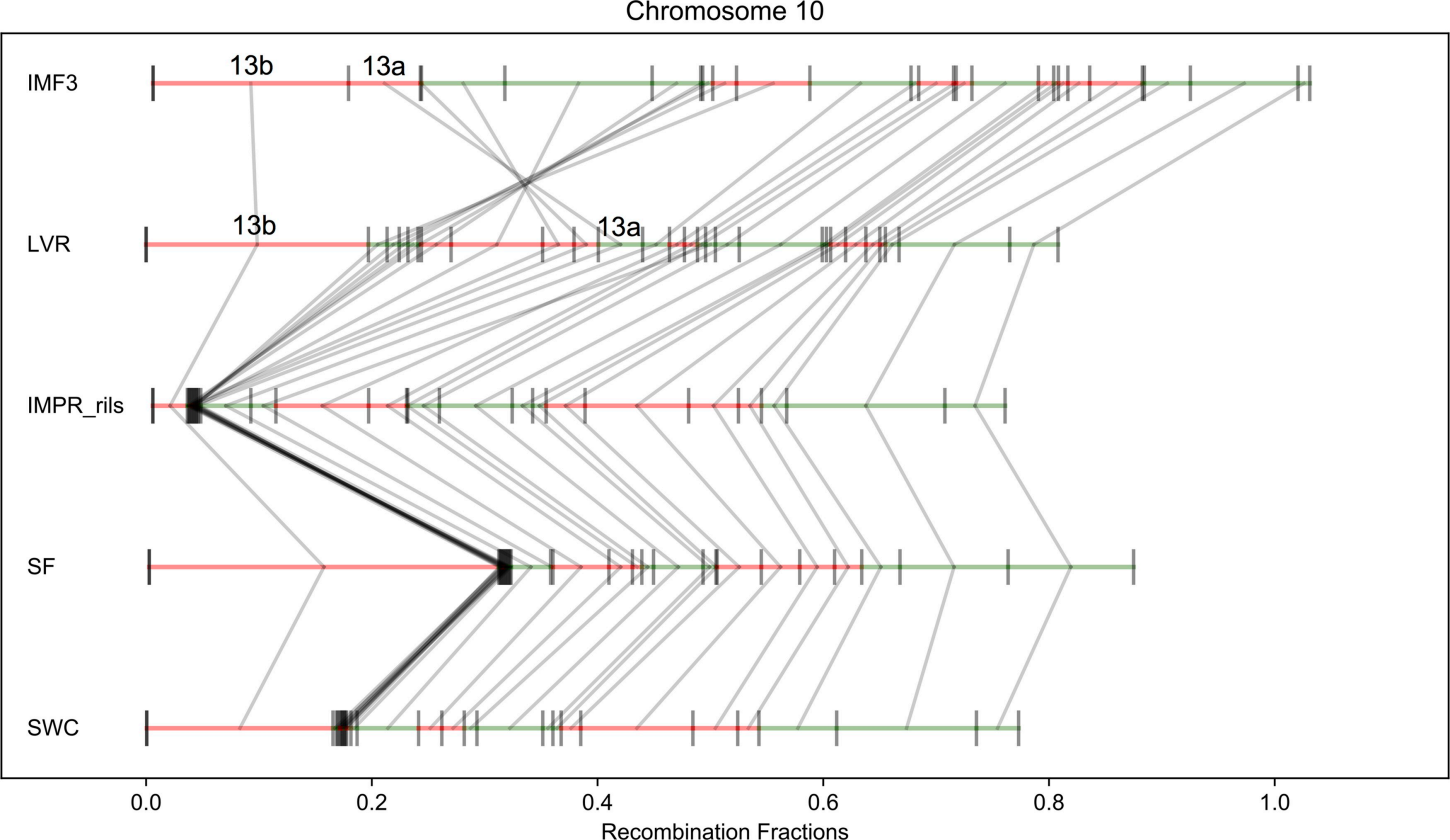
A reversal of genomic scaffolds due to an inversion is illustrated by the comparison of chromosome 10 maps for all five crosses. Each genomic scaffold is drawn to its genetic map length and denoted in green if it maps on the forward strand or red for the reverse strand. Grey lines connect the same scaffold between maps.

Our five mapping populations (Table 1) contain one intra-population cross (IMF3), two inter-population crosses (IMPR, IMSWC), a close interspecific-cross (IMNAS), and a more distant interspecific cross (DUNTIL). We observe structural polymorphisms by aligning the five maps to each other by chromosome. Fig 5 compares the maps for chromosome 10 which had previously been shown to harbor an inversion in IMPR [48].

As described in METHODS, we broke scaffold 13 into 13a and 13b based on a preliminary analysis of the DUNTIL data. GOOGA reassembled 13a and 13b into a continuous sequence for the IMF3 cross, but not the other crosses (Fig 5). There is minimal recombination between 13a and 13b in IMPR, IMNAS, and IMSWC because 13b is flanked by a large block of markers with nearly complete recombination suppression. This suppressed region, which represents at least 4.5 Mb of DNA, is freely recombining in IMF3 and DUNTIL but with a perfect reversal of marker order/orientation between those two crosses. From this, we infer that the IMF3 parents (IM62 and IM767; Table 1) each have inversion karyotype “A”, the DUNTIL parents (LVR and DUN) each have karyotype “B”, and the other three crosses are heterokaryotypic (one parent A and one B) for this inversion (S3 Fig). Noting that IM767 is orientation A, recombination suppression in the IMPR suggests the other parent in this cross (Point Reyes) has orientation B. By similar reasoning, we can conclude that SF5 and SWC also have orientation B (S3 Fig). The right half of chromosome 10 is largely collinear among all five crosses, indicating the inversion is the primary influence on chromosome-wide likelihood. The GOOGA lnLk of the IMF3 data is -3784. If the IMF3 data is forced into the optimized DUNTIL order, the lnLk drops to -4074 (ΔlnLk = 290; S5 Table). This gives strong statistical support of the inversion between the A and B homokarytypic crosses. The effect is less pronounced in the heterokaryotypic crosses. For example, the ΔlnLks of the IMNAS data when forced into the DUNTIL and IMF3 maps are 59 and 124, respectively (S5 Table). Thus, as expected, the recombination suppression in this heterokaryotypic cross results in relatively weak support for either a pure A or B inversion orientation.

The inversion on chromosome 10 is the only case among these crosses where we see free recombination in both homokaryotypes and suppression in the heterokaryotypes. In all other cases, one or more crosses reveal recombination suppression, with at least one homokaryotypic cross also present among our five populations (S11 Fig and S3 Table). Lowry and Willis [13] showed a reversal of marker order (as in Fig 5) for the inversion on chromosome 8. This feature is associated with annual versus perennial life-history within *M*. *guttatus*. Here, we see free recombination over the inverted region on chromosome 8 in IMF3, IMSWC, and IMNAS (annual x annual crosses), and suppression in IMPR and DUNTIL (annual x perennial *M*. *guttatus* and perennial *M*. *guttatus* x perennial *M*. *tilingii*, respectively). A similar pattern is noted for previously hypothesized inversions on chromosomes 5 (suppression in DUNTIL and IMPR) and 13 (suppression in DUNTIL) [49] and for the meiotic drive locus on chromosome 11 (suppression in IMF3) [63]. Given comparable evidence, we also identify three novel putative inversions (S2 Table). A region of at least 1.2 Mb spanning scaffolds 19, 73 and 65 on chromosome 2 is completely suppressed in the IMPR (S3 Fig). There is substantial recombination across this region in other crosses: 8 cM in DUNTIL, 10 cM in IMF3, 7 cM in IMNAS, and 2 cM in IMSWC. A larger physical region (≈5 Mb on chromosome 7) is fully suppressed in IMPR but not the other crosses (20–45 cM; S8 Fig). Finally, a stretch of ≈4 Mb on chromosome 14 is suppressed in the IMSWC cross but not in other crosses (about 30 cM, S15 Fig).

To provide a more general comparison of the extent of gene order differences among the crosses, we imposed the optimal map in every cross onto the genotypic data from every other cross and computed the ΔlnLk. Then we summed these for each chromosome. The larger this sum, the greater degree of structural discrepancy between maps for each cross (Table 2). As quantified by this metric, chromosome 10 has the greatest degree of structural discrepancy, an unsurprising result given the large polymorphic inversion (Fig 5). Chromosomes 5 and 11 are next, both of which show large tracts of reordered scaffolds and shared regions of recombination suppression among several crosses (Table 2). Surprisingly, the lowest value is for chromosome 8, which has a pairwise sum of ΔlnLk of only 75.4. The large inversion on chromosome 8 suppresses recombination in annual x perennial mapping populations, and as a consequence, the ordering of scaffolds within the inverted region is fairly arbitrary in those heterotypic crosses. There are few map changes for chromosome 8. This result arises because the strongly supported, co-linear maps from the homokaryotypic crosses (IMF3, IMSWC, and IMNAS) are also largely reiterated in the suppressed crosses (DUNTIL and IMPR). However, all GOOGA maps of chromosome 8 represent a vast improvement over the V2 map (mean ΔlnLk vs V2 = 985, S4 Table), suggesting the shared order that emerges from the inversion region is a large improvement over the reference genome.

**Table 2:** Summed pairwise changes in log-likelihood values by chromosome given in descending order. For each chromosome we imposed the final scaffold order from every cross onto every other cross and calculated the difference in the natural log likelihoods (ΔlnLk) between the crosses own order versus this imposed order. Sums are given on the set of ΔlnLks for each chromosome. A large positive ΔlnLk indicates a pair of crosses with highly incompatible orders.

Chromosome	Sum of Pairwise ΔlnLk
10	5581.1
5	5330.1
11	3503.0
3	2263.3
12	1449.2
13	1301.0
1	1203.7
7	845.8
6	828.2
2	782.0
4	610.8
14	551.0
9	442.1
8	75.4

### DNA composition predicts recombination rate variation in *Mimulus*

**Fig 6:**
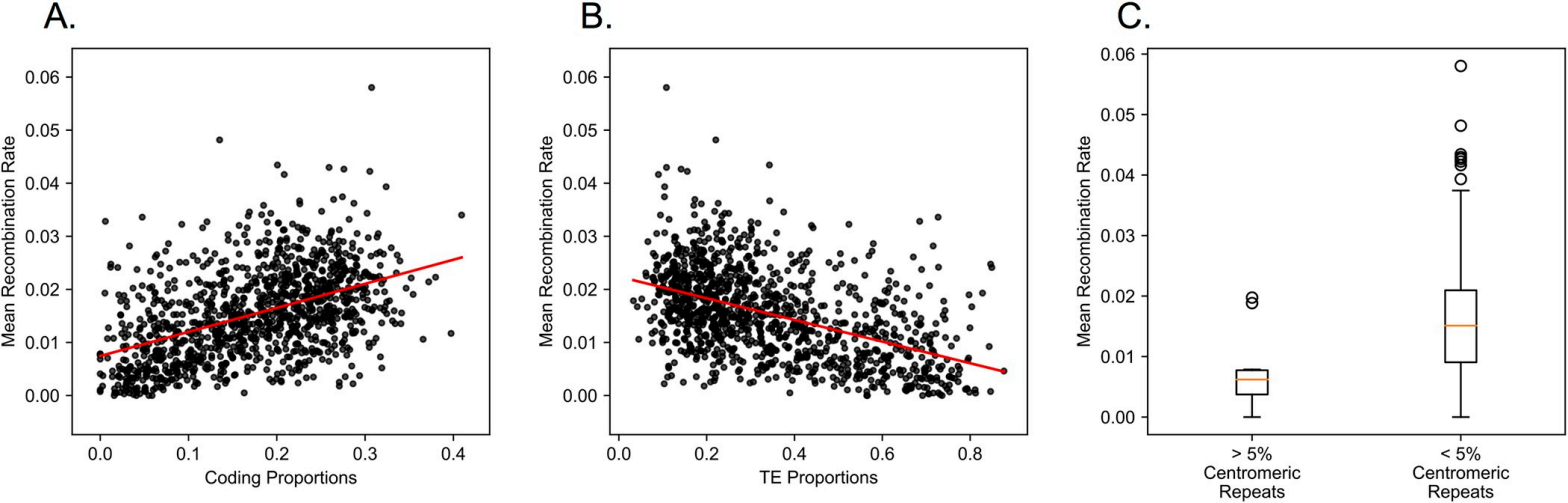
The effect of genomic features on recombination rate is illustrated. Panel A shows the positive correlation between the proportion of coding sequence and recombination rate. Panel B shows the negative correlation between the proportion annotated as a transposable element (TE) and recombination rate. For both panel A and B the red line markers the least squares best fit. Panel C shows the recombination rate distributions for 100 kb regions with >5% centromeric repeat content, or <5% centromeric repeat content.

We tested whether recombination rate is associated with the proportions of DNA annotated as coding sequence, transposable elements (TEs), and putative *M*. *guttatus* cent728 centromeric repeats [55] within a genomic window (Fig 6). Recombination rate is positively correlated with coding sequence density (Pearson’s r = 0.218) and is negatively correlated with TE density (Pearson’s r = -0.478). To test the impact of centromeric repeats [63] on recombination rate, we binned our 200 kb windows into those with < 5% centromeric repeat sequence vs. those with > 5%. Centromeres are expected to suppress recombination, and consistent with this prediction, we see a significant drop in recombination in windows with > 5% centromeric repeats (t-test p-value = 0.0003; Fig 6C). These results indicate that the recombination rates estimated by GOOGA fit well with biological expectations.

## Discussion

There has been a resurgence of interest among evolutionary biologists in structural variation, particularly in the contribution of chromosomal inversions to phenotypic variation, adaptive divergence among populations, and speciation [15–18, 64–68]. Inversions are routinely discovered from recombination suppression in genetic maps. They can be verified cytologically [11] or by reversal in marker order when comparing different genetic maps (e.g. [13], Fig 5). Our capacity to generate genetic maps has significantly advanced with RAD-seq and related genotyping platforms [34–38], particularly in non-model organisms. We developed GOOGA in response to these data, with an eye toward detecting structural variation. Accommodating error in map construction is particularly important with low-coverage sequencing and unpolished genome builds. Propagating genotype uncertainty throughout the process to the assignment of map likelihoods provides a means to determine how strongly the underlying genotype data support apparent differences in scaffold order and orientation. The application of GOOGA to detect previously identified structural polymorphism in *Mimulus* illustrates how evidence of map differences manifest as differences in likelihood (Figs 4 & 5).

Andolfatto et al. [36] developed an HMM for use with RAD-Seq like data in genetic mapping. We have adopted the HMM approach here, although with numerous updates to both the model and implementation. First, we define markers within genomic windows, each inclusive of many SNPs, e.g. [48]. SNP calls from closely linked sites are aggregated to make a putative call for the ancestry of each genomic region (e.g. AA, AB, or BB) of each recombinant individual. Given that the quality of individual DNA samples can vary greatly, we fit a model with individual specific genotyping error rates. The intervals between markers are treated differently depending on whether markers are within a genomic scaffold or between distinct scaffolds. The former are subject to tests for genotyping consistency given implied close linkage. The latter are explored using a genetic algorithm to order and orient scaffolds into chromosomes based on the likelihood of the data. Window-based genotype calling sacrifices resolution to obtain robust markers. In this application, we used 100 kb windows, which is about 0.3% of the average *Mimulus* chromosome. The resulting marker density is high relative to the number of recombination breakpoints per chromosome in F_2_, F_3_, and RIL individuals (typically 1–3; S3 Table) and sufficient for testing alternative maps. However, scoring recombination events at the scale of 100 kb does limit inference of genomic features that determine recombination. Factors defined effectively at the 100 kb scale, such as the density of genes or transposable elements exhibit clear correlations with recombination (Fig 6; [69]).

On the other hand, it is too coarse to evaluate finer scale determinants of recombination events. For example, population LD patterns suggest that recombination is strikingly elevated near the start site of genes in *M*. *guttatus* [46]. Fig 6A is fully consistent with this result: Mean recombination rate is correlated with the proportion of coding sequence, which is strongly correlated with number of gene start sites per window. However, at the resolution we selected, we cannot distinguish the effect of start sites from other features of gene rich regions.

The emission probabilities in the HMM portion of GOOGA are based on individualized genotyping error rates. This model feature stems from our observation that the quality of genotyping data varies quite substantially among samples, even of the same batch. This variability likely reflects stochastic factors, such as variation in the amount/quality of input DNA per individual. Regardless of cause, we find important differences in error rates among samples in all mapping populations (S1 Table), justifying the need to account for these error rates explicitly and individually. While rates are typically low in absolute terms (medians around 0.01 for e_0i_ and e_2i_, much smaller for e_1i_; see METHODS), they are not negligible relative to actual recombination rates between adjacent markers. Differences in genotyping error rates provide key weights on the contributions of different individuals to the overall likelihood of a map and its associated collection of recombination rate estimates. A practical example of the utility of genotype filtering and genotyping error estimation is that it enabled the discovery of two novel putative inversions in the IMPR cross (on chromosomes 2 and 7). These were not identified in the original paper [48] because those authors imposed conservative thresholds both on marker inclusion and on whether individuals were included in the final map construction. Twice as many of the plants from the IMPR population are included in the present analysis and 25% more of the genome is included in the map.

A diversity of factors may complicate marker construction from low-coverage sequencing data [55, 70, 71]. We implement filtering steps in GOOGA to mitigate these factors including SNP quality and allele frequency, SNP-level neighbor consistency tests, read-depth thresholds, marker-level (100 kb interval) neighbor consistency and heterozygosity tests, exclusion of individuals based on a high proportion of missing genotype calls, and/or high genotyping error rates. The goal of this filtering is to produce a marker set that is consistent with Mendelian segregation, but the filters will not always succeed. For example, a region on chromosome 5 involving only 7 markers in the IMF3 cross (corresponding to the small scaffolds 226, 252, 94, 368 and 358) contributes 97 cM to the map length (over 40% of the total for this chromosome). It is possible that this a high recombination region (an unknown amount of DNA resides between these scaffolds), but it seems more likely a spurious inflation. These scaffolds map inconsistently in the other crosses–if they appear at all, they are not always adjacent. Of course, incorrectly locating good markers can produce the same “map inflation” effect as properly locating misleading markers.

The simulation study (Fig 3) reinforces the limitations of using genetic map data to guide genome assembly. Even with fully accurate genotyping, recombination based methods cannot resolve non-recombining regions. Sequencing-based methods [72] may be required to identify structural variants in these situations. Additionally, as GOOGA requires genetic segregation data, it cannot be used in species where segregating families cannot be created experimentally or sampled from nature. We here analyzed standard designs (e.g. F_2_, F_3_, and RIL), but the HMM could be adapted to other situations such as outbred families or pedigrees. Finally, because GOOGA was designed to work with low-coverage sequence data, it requires several informative reads per windows, which may not be possible in species with extremely low genetic variation. However, outside of these exceptions, the GOOGA methodology is highly flexible and can be readily adapted to work in many species.

### Mimulus results

A tangible product of the application of GOOGA to *Mimulus guttatus* is that we substantially revise the reference genome of this species. The reference line (IM62) is used in two of our crosses, including in a cross to another line from the same population (IM767). Excepting the meiotic drive locus on chromosome 11 [63], IM767 appears to be largely collinear with IM62 (the two lines have the same orientation at other putative inversions). Despite this, the GOOGA realignment of scaffolds yields a dramatic increase in IMF3 likelihood over the V2 assembly: ΔlnLk is 5464 when summed over all chromosomes (S4 Table). Map revisions are found on each chromosome, and supported not only by ΔlnLk within IMF3, but also the maps from the other crosses. Incomplete assembly is a common and important problem in genomics, particularly in species with complex patterns of repeats. The promising implication of Fig 4 is that the rough genome assembly of many species can be dramatically improved with a low-coverage sequencing of a mapping population. Moreover, GOOGA quantifies the magnitude of improvements in terms of increase in likelihood.

The alignment of maps for *Mimulus* chromosome 10 illustrates the effect of an inversion. A 5 Mb region (left portion of Fig 5) was previously identified as a putative inversion from recombination suppression in the IMPR [48]. This is clearly confirmed here by the inclusion of both homokaryotypic crosses (AxA and BxB) and the ‘map flip’ (top two panels in Fig 5) effectively ascertains the scaffolds within the inversion and their ordering. This example also highlights the importance of marker construction. While it is possible to construct markers *de novo* with RAD-seq data [40], here we delineate markers on a previously assembled set of reference DNA sequences (the genomic scaffolds from the *M*. *guttatus* reference genome) obtained from sequencing of the IM62 inbred line. There are clear advantages to defining markers in this fashion, but care must be taken with this approach, especially in distantly related populations. For example, we initially assumed that the IM62 reference genome scaffolds correctly reflect the gene order for the other mapping populations. However, in our analysis of chromosome 10, we found it necessary to break scaffold 13 into two parts (13a and 13b), though it is continuous in IM62. GOOGA reannealed 13a and 13b in the IMF3 cross but inserted other scaffolds between them in other crosses due to a segregating inversion with a breakpoint contained within scaffold 13 (particularly DUNTIL; Fig 5). This implies that scaffold 13 was correctly assembled for IM62, but not for DUNTIL.

In the five *Mimulus* crosses, chromosome 10 is the only of the eight putative inversions where both homokaryotypic crosses are included. Reversal of marker ordering between homokaryotypic crosses was previously demonstrated for chromosome 8 [13], and could be demonstrated for others with appropriate selection of parental lines. However, the sort of reversal of genetic maps evident in Fig 5 requires polymorphism within both karyotypes. The derived karyotype is essentially homogeneous (few or no SNPs) for several structural polymorphisms in *M*. *guttatus* [12, 73]. Polymorphism should be evident within both karyotypes for inversions that have had time to accumulate variants through mutation or gene flux [74, 75], but perhaps not for very young inversions.

**Fig 7:**
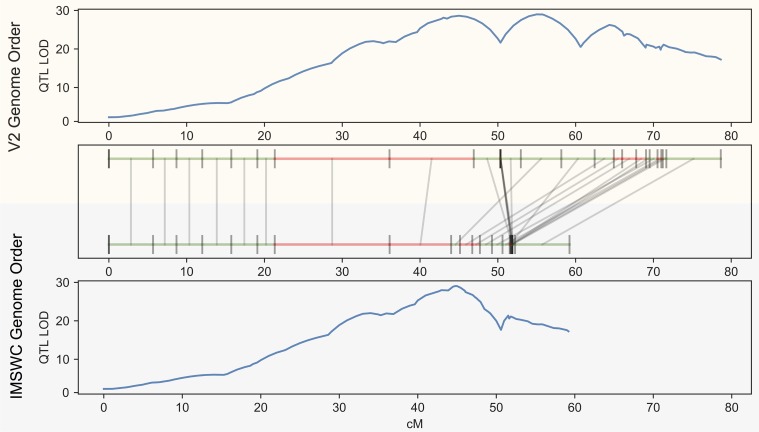
**The results of QTL estimation using the V2 map (top panel) versus the IMSWC optimized map from GOOGA (bottom panel).** The maps are aligned in middle panel. Each genomic scaffold is drawn to its genetic map length and denoted in green if it maps on the forward strand or red for the reverse strand. Grey lines connect the same scaffold between maps. QTL estimates are plotted for both panels as the log of the odds (LOD) score on the y-axis, and genetic map location on the x-axis.

The inclusion of phenotype data with the IMSWC cross illustrates the importance of scaffold ordering for downstream genetic analyses (Fig 7).

Here, each F_2_ plant was scored for progression to flowering, a dichotomous trait in the experimental photoperiod, which was restrictive to floral induction for one parent. Application of the same QTL mapping procedure to the data produces radically different outcomes if markers are placed according to the current *M*. *guttatus* reference genome (V2 map is the top panel of Fig 7) or by the GOOGA optimized map (bottom panel of Fig 7). Using the latter, which has a ΔlnLk improvement of 1089 over the V2 map (S4 Table), the data suggest a single large-effect QTL localized to a map position 44–45 cM into chromosome 11. QTL mapping to the V2 orientation yields three distinct peaks, each with a high LOD score. The specific markers near the QTL peak in the GOOGA map are jumbled in the V2 map which splits the genotype-phenotype association into three distinct parts. The V2 map is also “stretched”–expanded in recombination length by over 20 cM–likely to compensate for bad scaffold joins. Both of these effects impede QTL inference in places where the reference genome is misassembled.

### Application

The GOOGA pipeline is a set of modules (Fig 1): (A) procedures to make genetic markers from low-coverage sequencing data in conjunction with a collection of genomic scaffolds, (B) a method to estimate genotyping error rates specific to each individual, (C) an HMM to estimate recombination rates and obtain a likelihood for a specific ordering and orientation of genomic scaffolds, and (D) a genetic algorithm (GA) to search map space to obtain pseudo-chromosomes that maximize the likelihood of the data. While GOOGA was developed as an integrated series of steps, one or more of the components might be used apart from the rest. Below, we outline a few options that could be appropriate for different species or scenarios.

Most basically, one might use (A) to create markers and then apply standard map making software [76, 77] to the resulting genotype matrix. If this is done with the *Mimulus* mapping populations (or comparable datasets), the resulting matrices contain a great excess of missing data. As a consequence, extensive culling (both of individuals scored for too few markers and markers scored for too few individuals) is then required to apply standard map making programs. An alternative is to apply (A)-(B)-(C) to generate genetic markers. Assuming that the genomic scaffolds are generally reliable, the HMM will leverage data from neighboring windows to inform genotype calls. After obtaining the MLE on rates, one can extract posterior probabilities on genotypes and then impose ‘hard calls’, e.g. [41], to create a genotype matrix. We found this approach effective with as few as 10 informative reads per windows (see METHODS). Another possibility is to replace the front end of the pipeline. If one has high certainty in the validity of individual SNPs (their location and scoring), it is natural to replace windows (A) with individual SNPs as the observed states of the HMM ([36], Fig 2). Finally, while we found the GA effective for searching map space (D), other map optimization methods may prove useful in searching order/orientation space given that the HMM provides a likelihood for each (e.g., [78, 79]).

Like *Mimulus*, many species and species complexes harbor significant segregating inversions and other gene order polymorphisms including *Drosophila*, *Zea*, *Anopheles* and *Helianthus* [80–83]. The simulation study suggests a way that GOOGA could test whether sequences in a novel population under study are collinear with the reference genome. One could start by breaking the reference genome into ‘pseudo-scaffolds’ to be reassembled using data from the novel population. This is essentially what we did in the simulation experiment on *D*. *melanogaster* chromosome 2 (Fig 3). Sequences from recombinant individuals of the novel population would be mapped to the pseudo-scaffolds and the data input to GOOGA. Metrics like ΔlnLk can evaluate the reference genome map and identify necessary changes (as we have done in IMSWC in Fig 7). In this way, a necessary solution for species with incomplete genome assemblies (e.g. *Mimulus*) could be used in species with high-quality reference assemblies (e.g. *D*. *melanogaster*) but in populations that are structurally diverged from the reference genome. This application would be particularly convenient in cases where trait mapping is being performed via RAD-Seq genotyping, as no additional data would need to be generated.

## Supporting information

S1 Data Input data and software needed to simulate GOOGA inputs from *Drosophila*. This compressed .zip file includes genomic locations and recombination map used to simulate MSG like markers on simulated scaffolds (made by randomly breaking the genome assembly), and also Python programs use to perform these simulations. Running this code will produce inputs appropriate for GOOGA.
(ZIP)

S1 Fig The SNP level data is reported for each polymorphism within a particular scaffold in each of three plants of the IMSWC mapping panel. **At each SNP, the number of reads that match parent A and parent B are reported.** Each plant is heterozygous for their respective scaffold–there is extensive representation of both parents ***across SNPs***. However, a number of specific SNPs (color coded) exhibit strong bias towards one parent or the other (one allele is absent). These regions exhibit the usual tendency (typical of this mapping population and all the others) for allele counts to be over-dispersed relative to the binomial distribution. In other words, the fraction of SNPs that appear homozygous are much greater than expected if reads were sampled independently from heterozygous individual in library construction and sequencing. The graphs illustrate the mixed signal at the SNP level along each scaffold of each plant.
(XLSX)

S2 Fig Comparative genetic maps of *Mimulus* Chromosome 1 for each of the five populations used in this study. Each genomic scaffold is given in a separate color.
(PDF)

S3 Fig Comparative genetic maps of *Mimulus* Chromosome 2 for each of the five populations used in this study. Each genomic scaffold is given in a separate color.
(PDF)

S4 Fig Comparative genetic maps of *Mimulus* Chromosome 3 for each of the five populations used in this study. Each genomic scaffold is given in a separate color.
(PDF)

S5 Fig Comparative genetic maps of *Mimulus* Chromosome 4 for each of the five populations used in this study. Each genomic scaffold is given in a separate color.
(PDF)

S6 Fig Comparative genetic maps of *Mimulus* Chromosome 5 for each of the five populations used in this study. Each genomic scaffold is given in a separate color.
(PDF)

S7 Fig Comparative genetic maps of *Mimulus* Chromosome 6 for each of the five populations used in this study. Each genomic scaffold is given in a separate color.
(PDF)

S8 Fig Comparative genetic maps of *Mimulus* Chromosome 7 for each of the five populations used in this study. Each genomic scaffold is given in a separate color.
(PDF)

S9 Fig Comparative genetic maps of *Mimulus* Chromosome 8 for each of the five populations used in this study. Each genomic scaffold is given in a separate color.
(PDF)

S10 Fig Comparative genetic maps of *Mimulus* Chromosome 9 for each of the five populations used in this study. Each genomic scaffold is given in a separate color.
(PDF)

S11 Fig Comparative genetic maps of *Mimulus* Chromosome 10 for each of the five populations used in this study. Each genomic scaffold is given in a separate color.
(PDF)

S12 Fig Comparative genetic maps of *Mimulus* Chromosome 11 for each of the five populations used in this study. Each genomic scaffold is given in a separate color.
(PDF)

S13 Fig Comparative genetic maps of *Mimulus* Chromosome 12 for each of the five populations used in this study. Each genomic scaffold is given in a separate color.
(PDF)

S14 Fig Comparative genetic maps of *Mimulus* Chromosome 13 for each of the five populations used in this study. Each genomic scaffold is given in a separate color.
(PDF)

S15 Fig Comparative genetic maps of *Mimulus* Chromosome 14 for each of the five populations used in this study. Each genomic scaffold is given in a separate color.
(PDF)

S16 Fig Diagram of the inferences of inversion karyotypes in *Mimulus* chromosome 10. Chromosome diagrams for all five crosses are listed each in a separate panel, with each parent listed to the right of the diagram. The inversion karyotype is indicated by color (red or blue) and by either ‘>>>>>>>‘ or ‘<<<<<<<‘. The IMF3 and DUNTIL crosses were both formed by homokaryotypic parents and show free recombination. Thus these parents can be inferred as karyotypes A (IMF3) or B (DUNTIL). The next three crosses represent A/B heterokaryotypes and show strong recombination suppression. However, because parents from the IMF3 cross (A karyotype) were used in these crosses, we can infer that these parents are A and that the other must be B.
(PDF)

S1 Table Estimated genotype error rates for every individual from every population used in this study. (XLSX)

S2 Table All putative chromosomal inversions identified by GOOGA among the five mapping populations. (DOCX)

S3 Table This spreadsheet includes the maps of every chromosome for each cross, and physical and genetic location of each marker. The last marker of each chromosome gives the map lnLk.
(XLSX)

S4 Table Delta natural log likelihood (ΔlnLk) between GOOGA and V2 marker order. For each chromosome and each population the ΔlnLk is given. Large positive values indicate that the mapping data fits the GOOGA optimized order much better than the V2 genome order.
(XLSX)

S5 Table Likelihood differences from imposing each GOOGA optimized map order onto every other population. All pairwise contrasts between population are given for each chromosome. “Home” is the focal data set, and “away” indicates the imposed map order. ll1 is the likelihood of “home” in it’s own map order, and ll2 is “home” in the “away” order. “Diff” is the difference between ll1 and ll2.
(XLSX)

S6 Table Unplaced V2 genome scaffolds which were given a physical location from GOOGA. The table gives the v1 scaffold name and intervals for scaffolds that we were able to assign to one of the 14 *Mimulus* chromosomes. Their specific locations can be found in S1 Table.
(XLSX)

S1 Appendix Generating putative genotype calls in five Mimulus experiments. (DOCX)

S2 Appendix Model details. (DOCX)

S3 Appendix Genetic algorithm implementation. (DOCX)

## References

[1] Hirsch CN, Hirsch CD, Brohammer AB, Bowman MJ, Soifer I, Barad O, et al Draft assembly of elite inbred line PH207 provides insights into genomic and transcriptome diversity in maize. The Plant Cell. 2016;28(11):2700 10.1105/tpc.16.00353 27803309 PMC5155341

[2] Sudmant PH, Rausch T, Gardner EJ, Handsaker RE, Abyzov A, Huddleston J, et al An integrated map of structural variation in 2,504 human genomes. Nature. 2015;526(7571):75–81. 10.1038/nature15394 http://www.nature.com/nature/journal/v526/n7571/abs/nature15394.html#supplementary-information. 26432246 PMC4617611

[3] Feulner PGD, Chain FJJ, Panchal M, Eizaguirre C, Kalbe M, Lenz TL, et al Genome-wide patterns of standing genetic variation in a marine population of three-spined sticklebacks. Molecular Ecology. 2013;22(3):635–49. 10.1111/j.1365-294X.2012.05680.x 22747593

[4] Long Q, Rabanal FA, Meng D, Huber CD, Farlow A, Platzer A, et al Massive genomic variation and strong selection in Arabidopsis thaliana lines from Sweden. Nat Genet. 2013;45(8):884–90. 10.1038/ng.2678 http://www.nature.com/ng/journal/v45/n8/abs/ng.2678.html#supplementary-information. 23793030 PMC3755268

[5] Springer NM, Ying K, Fu Y, Ji T, Yeh C-T, Jia Y, et al Maize inbreds exhibit high levels of copy number variation (CNV) and presence/absence variation (PAV) in genome content. PLOS Genetics. 2009;5(11):e1000734 10.1371/journal.pgen.1000734 19956538 PMC2780416

[6] Li Y-h, Zhou G, Ma J, Jiang W, Jin L-g, Zhang Z, et al De novo assembly of soybean wild relatives for pan-genome analysis of diversity and agronomic traits. Nat Biotech. 2014;32(10):1045–52. 10.1038/nbt.2979 http://www.nature.com/nbt/journal/v32/n10/abs/nbt.2979.html#supplementary-information. 25218520

[7] Wright KM, Hellsten U, Xu C, Jeong AL, Sreedasyam A, Chapman JA, et al Adaptation to heavy-metal contaminated environments proceeds via selection on pre-existing genetic variation. bioRxiv. 2015 10.1101/029900

[8] Naseeb S, Carter Z, Minnis D, Donaldson I, Zeef L, Delneri D. Widespread Impact of Chromosomal Inversions on Gene Expression Uncovers Robustness via Phenotypic Buffering. Molecular Biology and Evolution. 2016;33(7):1679–96. 10.1093/molbev/msw045 26929245 PMC4915352

[9] Coluzzi M, Sabatini A, della Torre A, Di Deco MA, Petrarca V. A Polytene Chromosome Analysis of the Anopheles gambiae Species Complex. Science. 2002;298(5597):1415–8. 10.1126/science.1077769 12364623

[10] Fang Z, Pyhäjärvi T, Weber AL, Dawe RK, Glaubitz JC, Sánchez González JdJ, et al Megabase-scale inversion polymorphism in the wild ancestor of maize. Genetics. 2012 10.1534/genetics.112.138578 22542971 PMC3389981

[11] Krimbas CB, Powell JR. Drosophila Inversion Polymorphism Boca Raton, FL: CRC Press; 1992.

[12] Lee YW, Fishman L, Kelly JK, Willis JH. A Segregating Inversion Generates Fitness Variation in Yellow Monkeyflower (Mimulus guttatus). Genetics. 2016;202(4):1473–84. 10.1534/genetics.115.183566 26868767 PMC4905537

[13] Lowry DB, Willis JH. A Widespread Chromosomal Inversion Polymorphism Contributes to a Major Life-History Transition, Local Adaptation, and Reproductive Isolation. PLoS Biol. 2010;8(9):e1000500 10.1371/journal.pbio.1000500 20927411 PMC2946948

[14] Stankiewicz P, Lupski JR. Structural variation in the human genome and its role in disease. Annual Review of Medicine. 2010;61(1):437–55. 10.1146/annurev-med-100708-204735 20059347

[15] Schwander T, Libbrecht R, Keller L. Supergenes and Complex Phenotypes. Current Biology. 2014;24(7):R288–R94. 10.1016/j.cub.2014.01.056 24698381

[16] Love RR, Steele AM, Coulibaly MB, Traore SF, Emrich SJ, Fontaine MC, et al Chromosomal inversions and ecotypic differentiation in Anopheles gambiae: the perspective from whole-genome sequencing. Molecular Ecology. 2016;25(23):5889–906. 10.1111/mec.13888 27759895 PMC5130611

[17] Dagilis AJ, Kirkpatrick M. Prezygotic isolation, mating preferences, and the evolution of chromosomal inversions. Evolution. 2016;70(7):1465–72. 10.1111/evo.12954 27174252

[18] Samuk K. Inversions and the origin of behavioral differences in cod. Molecular Ecology. 2016;25(10):2111–3. 10.1111/mec.13624 27213696

[19] Fransz P, Linc G, Lee C-R, Aflitos SA, Lasky JR, Toomajian C, et al Molecular, genetic and evolutionary analysis of a paracentric inversion in *Arabidopsis thaliana*. The Plant Journal. 2016;88(2):159–78. 10.1111/tpj.13262 27436134 PMC5113708

[20] Hufford MB, Xu X, van Heerwaarden J, Pyhajarvi T, Chia J-M, Cartwright RA, et al Comparative population genomics of maize domestication and improvement. Nat Genet. 2012;44(7):808–11. 10.1038/ng.2309 http://www.nature.com/ng/journal/v44/n7/abs/ng.2309.html#supplementary-information. 22660546 PMC5531767

[21] Pyhäjärvi T, Hufford MB, Mezmouk S, Ross-Ibarra J. Complex Patterns of Local Adaptation in Teosinte. Genome Biology and Evolution. 2013;5(9):1594–609. 10.1093/gbe/evt109 23902747 PMC3787665

[22] Long A, Liti G, Luptak A, Tenaillon O. Elucidating the molecular architecture of adaptation via evolve and resequence experiments. Nature Reviews Genetics. 2015;16:567 10.1038/nrg3937 https://www.nature.com/articles/nrg3937#supplementary-information. 26347030 PMC4733663

[23] Nuzhdin SV, Turner TL. Promises and limitations of hitchhiking mapping. Current Opinion in Genetics & Development. 2013;23(6):694–9. 10.1016/j.gde.2013.10.002. 24239053 PMC3872824

[24] Kelly JK, Koseva B, Mojica JP. The Genomic Signal of Partial Sweeps in Mimulus guttatus. Genome Biology and Evolution. 2013;5(8):1457–69. 10.1093/gbe/evt100 23828880 PMC3762192

[25] Beissinger TM, Hirsch CN, Vaillancourt B, Deshpande S, Barry K, Buell CR, et al A Genome-Wide Scan for Evidence of Selection in a Maize Population Under Long-Term Artificial Selection for Ear Number. Genetics. 2014;196(3):829–40. 10.1534/genetics.113.160655 PMC3948809. 24381334 PMC3948809

[26] Beissinger TM, Rosa GJM, Kaeppler SM, Gianola D, de Leon N. Defining window-boundaries for genomic analyses using smoothing spline techniques. Genetics Selection Evolution. 2015;47(1):30 10.1186/s12711-015-0105-9 25928167 PMC4404117

[27] Chen M, Presting G, Barbazuk WB, Goicoechea JL, Blackmon B, Fang G, et al An Integrated Physical and Genetic Map of the Rice Genome. The Plant Cell. 2002;14(3):537–45. 10.1105/tpc.010485 11910002 PMC150577

[28] Staňková H, Hastie AR, Chan S, Vrána J, Tulpová Z, Kubaláková M, et al BioNano genome mapping of individual chromosomes supports physical mapping and sequence assembly in complex plant genomes. Plant Biotechnology Journal. 2016;14(7):1523–31. 10.1111/pbi.12513 26801360 PMC5066648

[29] Verde I, Jenkins J, Dondini L, Micali S, Pagliarani G, Vendramin E, et al The Peach v2.0 release: high-resolution linkage mapping and deep resequencing improve chromosome-scale assembly and contiguity. BMC Genomics. 2017;18(1):225 10.1186/s12864-017-3606-9 28284188 PMC5346207

[30] (ICGMC) ICGMC. High-Resolution Linkage Map and Chromosome-Scale Genome Assembly for Cassava (Manihot esculenta Crantz) from 10 Populations. G3: Genes|Genomes|Genetics. 2015;5(1):133–44. 10.1534/g3.114.015008 25504737 PMC4291464

[31] Hahn MW, Zhang SV, Moyle LC. Sequencing, assembling, and correcting draft genomes using recombinant populations. G3. 2014;4(4):669–79. 10.1534/g3.114.010264 24531727 PMC4059239

[32] Mascher M, Muehlbauer GJ, Rokhsar DS, Chapman J, Schmutz J, Barry K, et al Anchoring and ordering NGS contig assemblies by population sequencing (POPSEQ). The Plant Journal. 2013;76(4):718–27. 10.1111/tpj.12319 23998490 PMC4298792

[33] Nossa CW, Havlak P, Yue J-X, Lv J, Vincent KY, Brockmann HJ, et al Joint assembly and genetic mapping of the Atlantic horseshoe crab genome reveals ancient whole genome duplication. GigaScience. 2014;3(1):1–21. 10.1186/2047-217X-3-1 24987520 PMC4066314

[34] Davey JW, Blaxter ML. RADSeq: next-generation population genetics. Briefings in Functional Genomics. 2010;9(5–6):416–23. 10.1093/bfgp/elq031 PMC3080771. 21266344 PMC3080771

[35] Van Tassell CP, Smith TPL, Matukumalli LK, Taylor JF, Schnabel RD, Lawley CT, et al SNP discovery and allele frequency estimation by deep sequencing of reduced representation libraries. Nat Meth. 2008;5(3):247–52. http://www.nature.com/nmeth/journal/v5/n3/suppinfo/nmeth.1185_S1.html. 10.1038/nmeth.1185 18297082

[36] Andolfatto P, Davison D, Erezyilmaz D, Hu TT, Mast J, Sunayama-Morita T, et al Multiplexed shotgun genotyping for rapid and efficient genetic mapping. Genome research. 2011;21(4):610–7. 10.1101/gr.115402.110 WOS:000289067800011. 21233398 PMC3065708

[37] Elshire RJ, Glaubitz JC, Sun Q, Poland JA, Kawamoto K, Buckler ES, et al A Robust, Simple Genotyping-by-Sequencing (GBS) Approach for High Diversity Species. PLOS ONE. 2011;6(5):e19379 10.1371/journal.pone.0019379 21573248 PMC3087801

[38] Huang X, Feng Q, Qian Q, Zhao Q, Wang L, Wang A, et al High-throughput genotyping by whole-genome resequencing. Genome Research. 2009;19(6):1068–76. 10.1101/gr.089516.108 19420380 PMC2694477

[39] Eaton DAR. PyRAD: assembly of de novo RADseq loci for phylogenetic analyses. Bioinformatics. 2014;30(13):1844–9. 10.1093/bioinformatics/btu121 24603985

[40] Catchen J, Hohenlohe PA, Bassham S, Amores A, Cresko WA. Stacks: an analysis tool set for population genomics. Molecular ecology. 2013;22(11):3124–40. 10.1111/mec.12354 PMC3936987. 23701397 PMC3936987

[41] Slotte T, Hazzouri KM, Stern D, Andolfatto P, Wright SI. GENETIC ARCHITECTURE AND ADAPTIVE SIGNIFICANCE OF THE SELFING SYNDROME IN CAPSELLA. Evolution. 2012;66(5):1360–74. 10.1111/j.1558-5646.2011.01540.x WOS:000303047300006. 22519777 PMC5063048

[42] Flagel LE, Willis JH, Vision TJ. The standing pool of genomic structural variation in a natural population of Mimulus guttatus. Genome biology and evolution. 2014;6(1):53–64. 10.1093/gbe/evt199 24336482 PMC3914686

[43] Brandvain Y, Kenney AM, Flagel L, Coop G, Sweigart AL. Speciation and Introgression between *Mimulus nasutus* and *Mimulus guttatus*. PLOS Genetics. 2014;10(6):e1004410 10.1371/journal.pgen.1004410 24967630 PMC4072524

[44] Puzey JR, Willis JH, Kelly JK. Population structure and local selection yield high genomic variation in Mimulus guttatus. Molecular Ecology. 2017;26(2):519–35. 10.1111/mec.13922 27859786 PMC5274581

[45] Vickery RK. Case studies in the evolution of species complexes in Mimulus. Evol Biol 1978;11:405–507.

[46] Hellsten U, Wright KM, Jenkins J, Shu S, Yuan Y, Wessler SR, et al Fine-scale variation in meiotic recombination in Mimulus inferred from population shotgun sequencing. Proc Natl Acad Sci. 2013;110 10.1073/pnas.1319032110 24225854 PMC3845195

[47] Fishman L, Kelly AJ, Morgan E, Willis JH. A genetic map in the Mimulus guttatus species complex reveals transmission ratio distortion due to heterospecific interactions. Genetics. 2001;159(4):1701–16. PMC1461909. 11779808 10.1093/genetics/159.4.1701 PMC1461909

[48] Holeski L, Monnahan P, Koseva B, McCool N, Lindroth RL, Kelly JK. A High-Resolution Genetic Map of Yellow Monkeyflower Identifies Chemical Defense QTLs and Recombination Rate Variation. G3: Genes|Genomes|Genetics. 2014;4(5):813–21. 10.1534/g3.113.010124 24626287 PMC4025480

[49] Garner AG, Kenney AM, Fishman L, Sweigart AL. Genetic loci with parent-of-origin effects cause hybrid seed lethality in crosses between Mimulus species. New Phytologist. 2016;211(1):319–31. 10.1111/nph.13897 26924810

[50] Fishman L, Willis JH, Wu CA, Lee YW. Comparative linkage maps suggest that fission, not polyploidy, underlies near-doubling of chromosome number within monkeyflowers (Mimulus; Phrymaceae). Heredity. 2014;112(5):562–8. 10.1038/hdy.2013.143 24398885 PMC3998785

[51] Chakraborty M, VanKuren NW, Zhao R, Zhang X, Kalsow S, Emerson JJ. Hidden genetic variation shapes the structure of functional elements in Drosophila. Nature Genetics. 2018;50(1):20–5. 10.1038/s41588-017-0010-y 29255259 PMC5742068

[52] Hackett JL, Wang X, Smith BR, Macdonald SJ. Mapping QTL Contributing to Variation in Posterior Lobe Morphology between Strains of Drosophila melanogaster. PLOS ONE. 2016;11(9):e0162573 10.1371/journal.pone.0162573 27606594 PMC5015897

[53] Comeron JM, Ratnappan R, Bailin S. The Many Landscapes of Recombination in Drosophila melanogaster. PLOS Genetics. 2012;8(10):e1002905 10.1371/journal.pgen.1002905 23071443 PMC3469467

[54] McKenna A, Hanna M, Banks E, Sivachenko A, Cibulskis K, Kernytsky A, et al The Genome Analysis Toolkit: A MapReduce framework for analyzing next-generation DNA sequencing data. Genome Research. 2010;20(9):1297–303. 10.1101/gr.107524.110 20644199 PMC2928508

[55] Monnahan PJ, Colicchio J, Kelly JK. A genomic selection component analysis characterizes migration-selection balance. Evolution. 2015;69(7):1713–27. 10.1111/evo.12698 26082096 PMC4774264

[56] Wessinger CA, Kelly JK, Jiang P, Rausher MD, Hileman LC. SNP-skimming: A fast approach to map loci generating quantitative variation in natural populations. Molecular Ecology Resources. 0(0). 10.1111/1755-0998.12930 30033616 PMC6207453

[57] Broman KW, Sen S. A Guide to QTL Mapping with R/qtl. New York: Springer-Verlag; 2009.

[58] King EG, Macdonald SJ, Long AD. Properties and Power of the Drosophila Synthetic Population Resource for the Routine Dissection of Complex Traits. Genetics. 2012;191(3):935–49. 10.1534/genetics.112.138537 22505626 PMC3389985

[59] Zamanzad GF, Jürgen C, Tomasz B. A Nonhomogeneous Hidden Markov Model for Gene Mapping Based on Next-Generation Sequencing Data. Journal of Computational Biology. 2015;22(2):178–88. 10.1089/cmb.2014.0258 . 25611462

[60] Rabiner LR, Juang BH. An introduction to hidden Markov models. IEEE ASSP Magazine. 1986;1:4–15.

[61] Byrd RH, Lu P, Nocedal J. A Limited Memory Algorithm for Bound Constrained Optimization. SIAM Journal on Scientific and Statistical Computing. 1995;16(5):1190–208.

[62] Holland JH. Adaptation in natural and artificial systems: an introductory analysis with applications to biology, control, and artificial intelligence. Cambridge, Massachusetts: MIT Press; 1992.

[63] Fishman L, Saunders A. Centromere-Associated Female Meiotic Drive Entails Male Fitness Costs in Monkeyflowers. Science. 2008;322(5907):1559–62. 10.1126/science.1161406 19056989

[64] Tuttle Elaina, Bergland Alan O, Korody Marisa L, Brewer Michael S, Newhouse Daniel J, Minx P, et al Divergence and Functional Degradation of a Sex Chromosome-like Supergene. Current Biology. 2016;26(3):344–50. 10.1016/j.cub.2015.11.069 26804558 PMC4747794

[65] Feulner PGD, De-Kayne R. Genome evolution, structural rearrangements and speciation. Journal of Evolutionary Biology. 2017;30(8):1488–90. 10.1111/jeb.13101 28786195

[66] Wellenreuther M, Rosenquist H, Jaksons P, Larson KW. Local adaptation along an environmental cline in a species with an inversion polymorphism. Journal of Evolutionary Biology. 2017;30(6):1068–77. 10.1111/jeb.13064 28295810

[67] Kozak GM, Wadsworth CB, Kahne SC, Bogdanowicz SM, Harrison RG, Coates BS, et al A combination of sexual and ecological divergence contributes to rearrangement spread during initial stages of speciation. Molecular Ecology. 2017;26(8):2331–47. 10.1111/mec.14036 28141898

[68] Santos J, Pascual M, Fragata I, Simões P, Santos MA, Lima M, et al Tracking changes in chromosomal arrangements and their genetic content during adaptation. Journal of Evolutionary Biology. 2016;29(6):1151–67. 10.1111/jeb.12856 26969850

[69] Kent TV, Uzunović J, Wright SI. Coevolution between transposable elements and recombination. Philosophical Transactions of the Royal Society B: Biological Sciences. 2017;372(1736). 10.1098/rstb.2016.0458 29109221 PMC5698620

[70] Gautier M, Gharbi K, Cezard T, Foucaud J, Kerdelhué C, Pudlo P, et al The effect of RAD allele dropout on the estimation of genetic variation within and between populations. Molecular Ecology. 2013;22(11):3165–78. 10.1111/mec.12089 23110526

[71] Davey JW, Cezard T, Fuentes-Utrilla P, Eland C, Gharbi K, Blaxter ML. Special features of RAD Sequencing data: implications for genotyping. Molecular Ecology. 2013;22(11):3151–64. 10.1111/mec.12084 23110438 PMC3712469

[72] Corbett-Detig RB, Cardeno C, Langley CH. Sequence-Based Detection and Breakpoint Assembly of Polymorphic Inversions. Genetics. 2012;192(1):131–7. 10.1534/genetics.112.141622 22673805 PMC3430529

[73] Fishman L, Kelly JK. Centromere-associated meiotic drive and female fitness variation in Mimulus. Evolution. 2015;69(5):1208–18. 10.1111/evo.12661 25873401 PMC5958614

[74] Pegueroles C, Aquadro CF, Mestres F, Pascual M. Gene flow and gene flux shape evolutionary patterns of variation in Drosophila subobscura. Heredity. 2013;110:520 10.1038/hdy.2012.118 https://www.nature.com/articles/hdy2012118#supplementary-information. 23321709 PMC3656635

[75] Navarro A, Betrán E, Barbadilla A, Ruiz A. Recombination and Gene Flux Caused by Gene Conversion and Crossing Over in Inversion Heterokaryotypes. Genetics. 1997;146(2):695–709. 9178017 10.1093/genetics/146.2.695 PMC1208008

[76] Arends D, Prins P, Jansen RC, Broman KW. R/qtl: high-throughput multiple QTL mapping. Bioinformatics. 2010;26(23):2990–2. 10.1093/bioinformatics/btq565 20966004 PMC2982156

[77] Iwata H, Ninomiya S. AntMap: Constructing genetic linkage maps using an ant colony optimization algorithm. Breeding science. 2006;56:371–7.

[78] Green PJ. Reversible jump Markov chain Monte Carlo computation and Bayesian model determination. Biometrika. 1995;82(4):711–32. 10.1093/biomet/82.4.711

[79] Tang J, Moret BME. Scaling up accurate phylogenetic reconstruction from gene-order data. Bioinformatics. 2003;(Eleventh International Conference on Intelligent Systems for Molecular Biology). 10.1093/bioinformatics/btg1042 12855474

[80] Corbett-Detig RB, Hartl DL. Population Genomics of Inversion Polymorphisms in Drosophila melanogaster. PLOS Genetics. 2012;8(12):e1003056 10.1371/journal.pgen.1003056 23284285 PMC3527211

[81] Ayala D, Ullastres A, González J. Adaptation through chromosomal inversions in Anopheles. Frontiers in Genetics. 2014;5(129). 10.3389/fgene.2014.00129 24904633 PMC4033225

[82] Barb JG, Bowers JE, Renaut S, Rey JI, Knapp SJ, Rieseberg LH, et al Chromosomal Evolution and Patterns of Introgression in Helianthus. Genetics. 2014;197(3):969–79. 10.1534/genetics.114.165548 24770331 PMC4096374

[83] Fang Z, Pyhäjärvi T, Weber AL, Dawe RK, Glaubitz JC, González JdJS, et al Megabase-Scale Inversion Polymorphism in the Wild Ancestor of Maize. Genetics. 2012;191(3):883–94. 10.1534/genetics.112.138578 22542971 PMC3389981

