# Supplementary figures and images for: GOOGA: A platform to synthesize mapping experiments and identify genomic structural diversity
Source: PLoS Comput Biol. 2019 Apr 15;15(4):e1006949. doi: 10.1371/journal.pcbi.1006949 (PMC6483263; doi:10.1371/journal.pcbi.1006949)

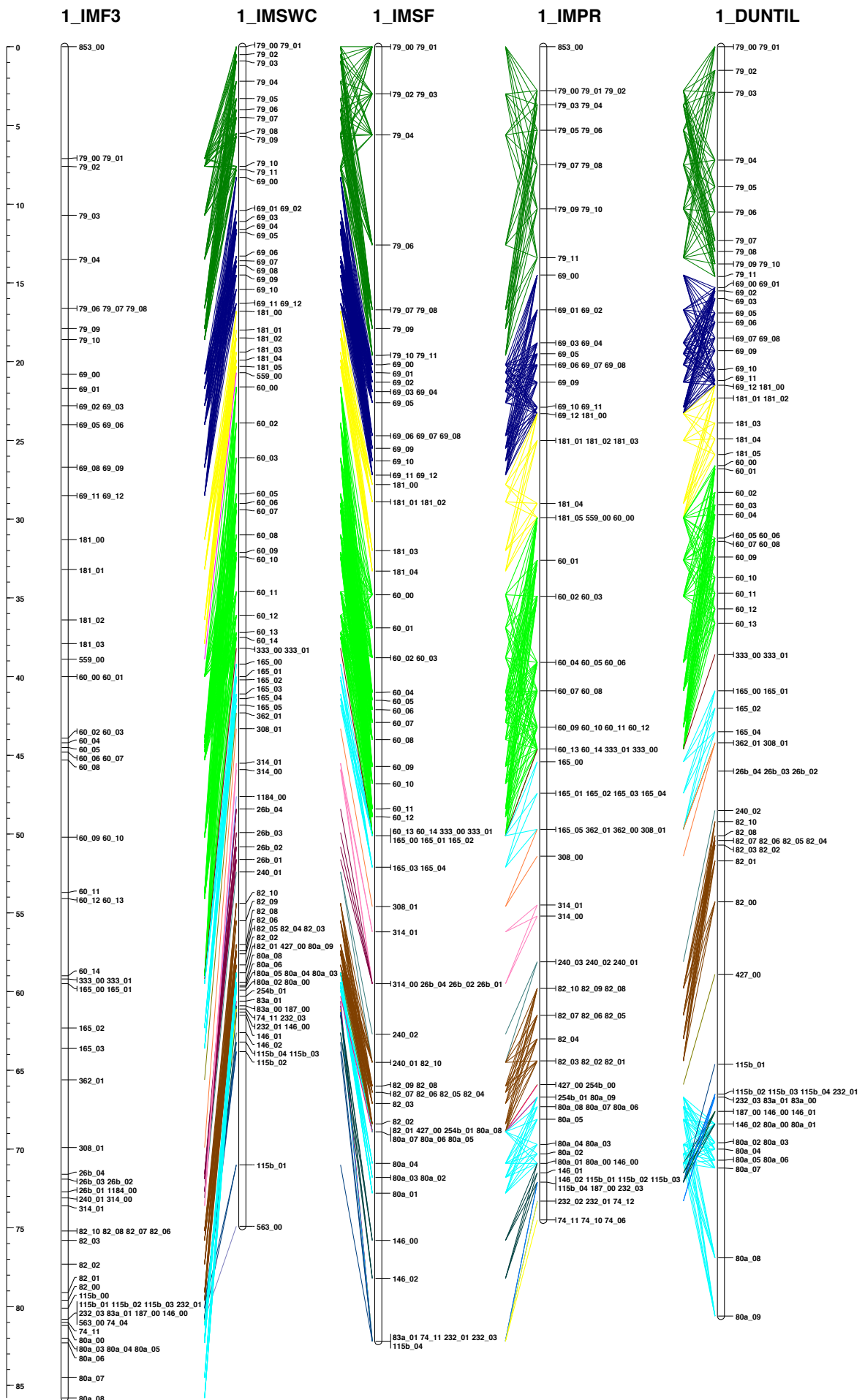

Supplement: S2 Fig — Each genomic scaffold is given in a separate color. (PDF) [file pcbi.1006949.s003.pdf]

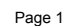

Supplement: S3 Fig — Each genomic scaffold is given in a separate color. (PDF) [file pcbi.1006949.s004.pdf]

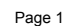

Supplement: S4 Fig — Each genomic scaffold is given in a separate color. (PDF) [file pcbi.1006949.s005.pdf]

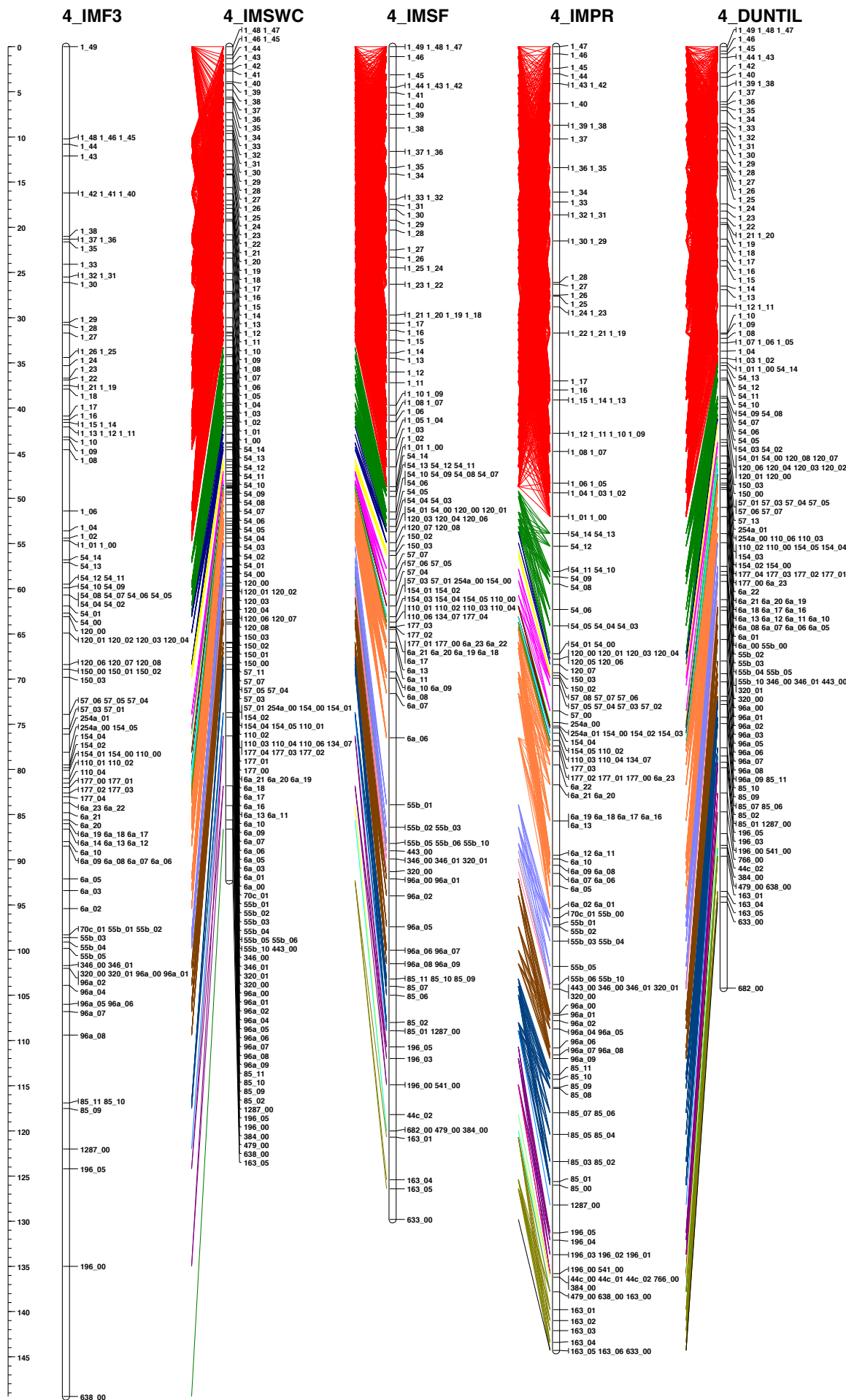

Supplement: S5 Fig — Each genomic scaffold is given in a separate color. (PDF) [file pcbi.1006949.s006.pdf]

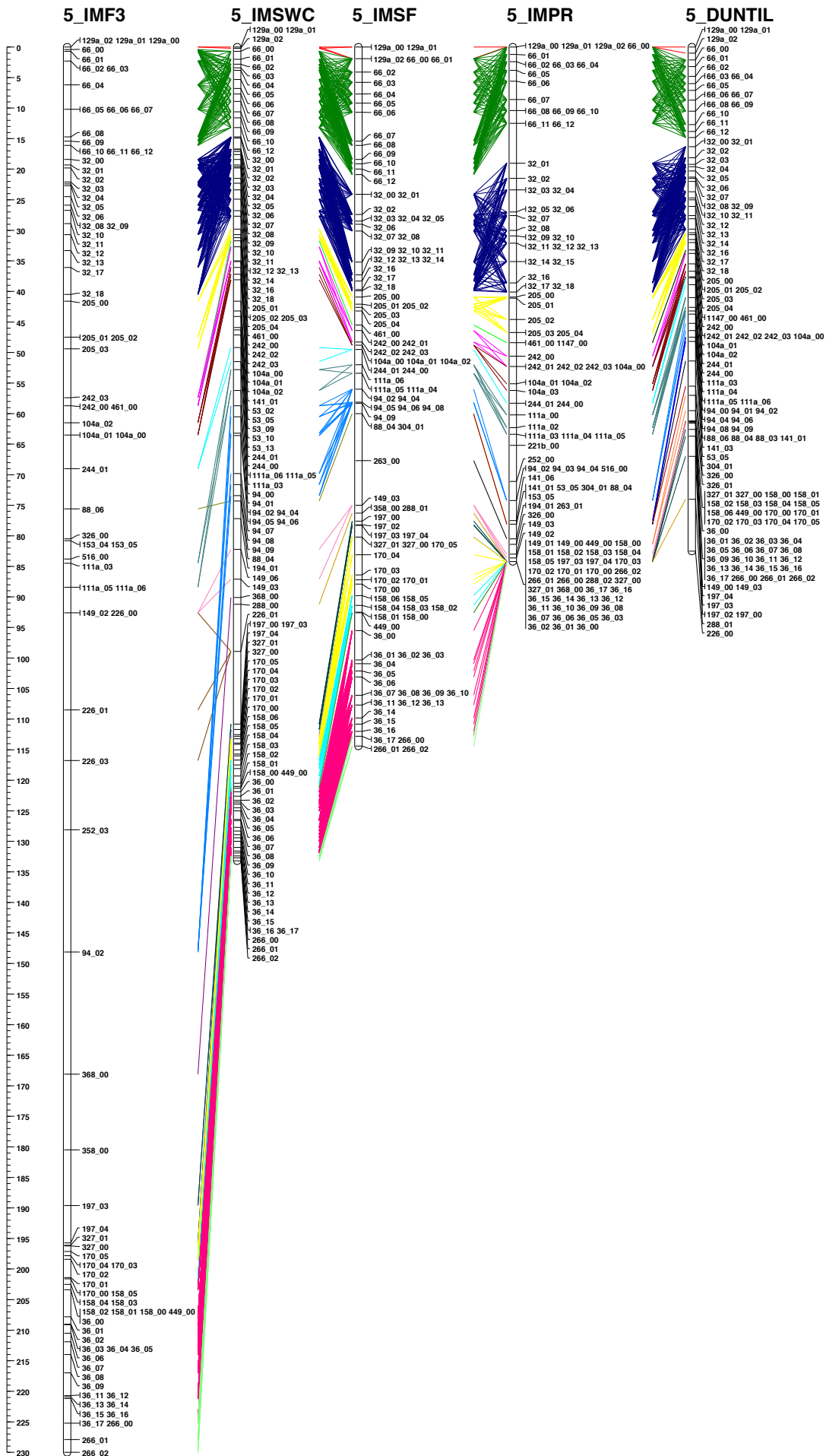

Supplement: S6 Fig — Each genomic scaffold is given in a separate color. (PDF) [file pcbi.1006949.s007.pdf]

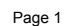

Supplement: S7 Fig — Each genomic scaffold is given in a separate color. (PDF) [file pcbi.1006949.s008.pdf]

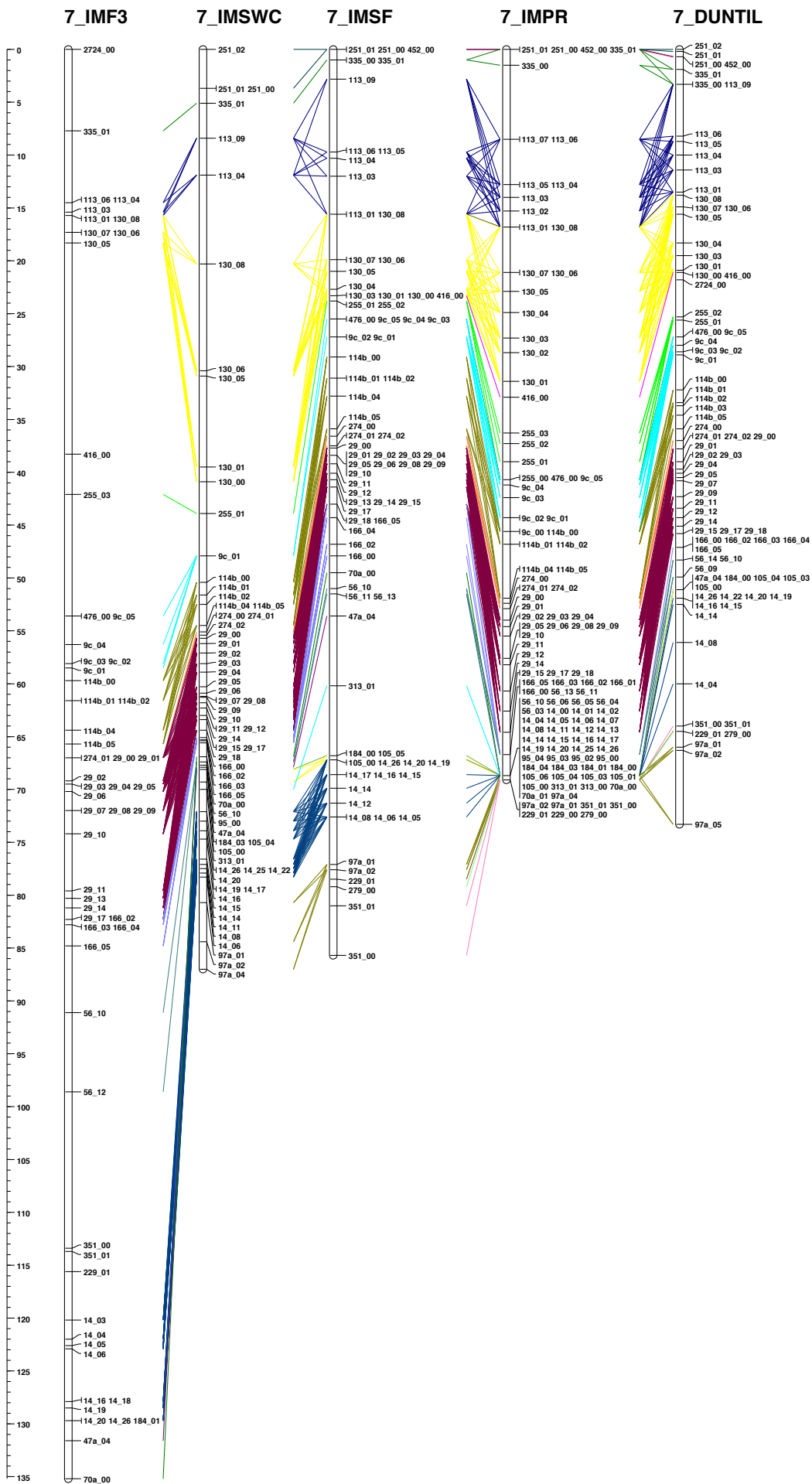

Supplement: S8 Fig — Each genomic scaffold is given in a separate color. (PDF) [file pcbi.1006949.s009.pdf]

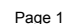

Supplement: S9 Fig — Each genomic scaffold is given in a separate color. (PDF) [file pcbi.1006949.s010.pdf]

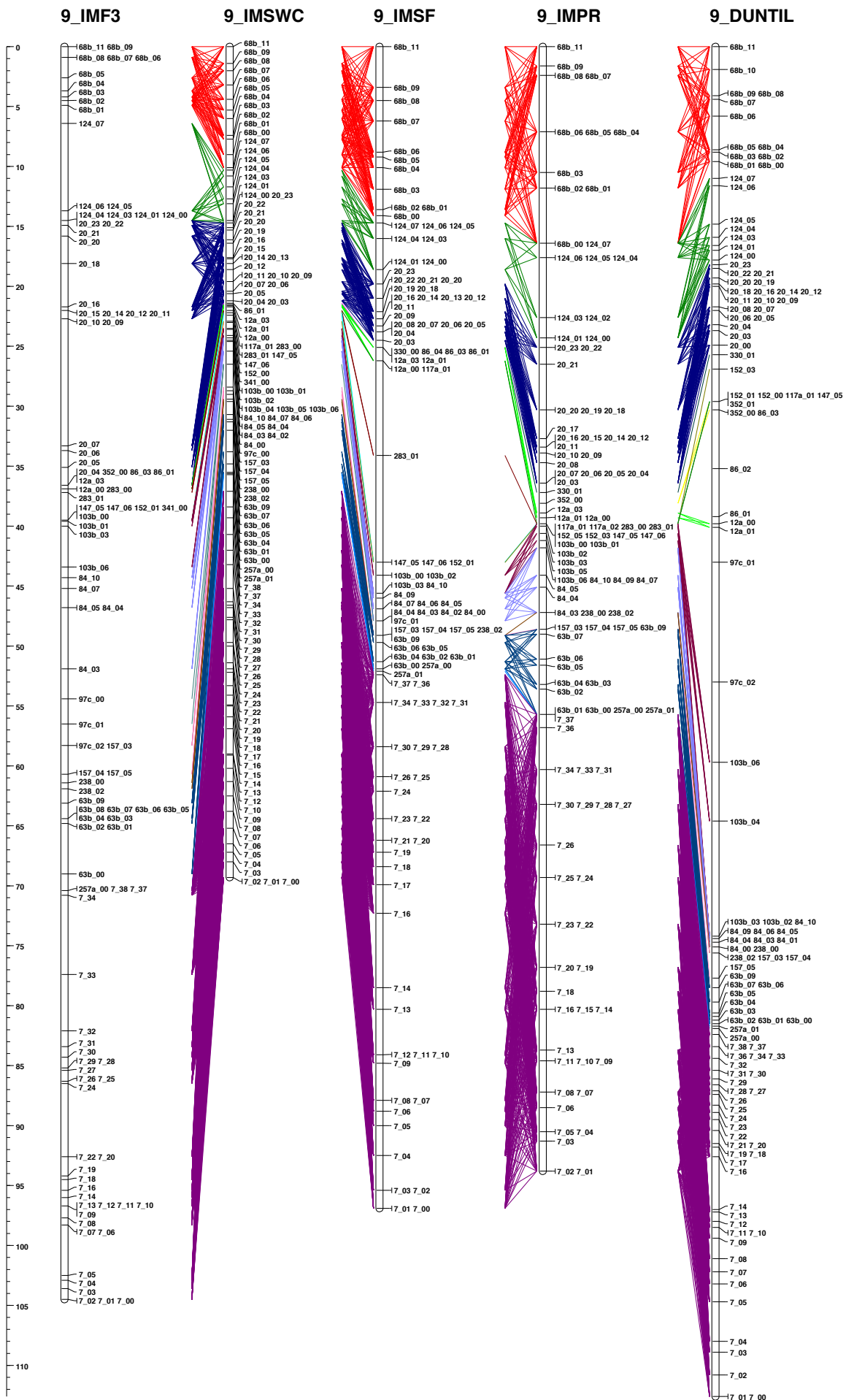

Supplement: S10 Fig — Each genomic scaffold is given in a separate color. (PDF) [file pcbi.1006949.s011.pdf]

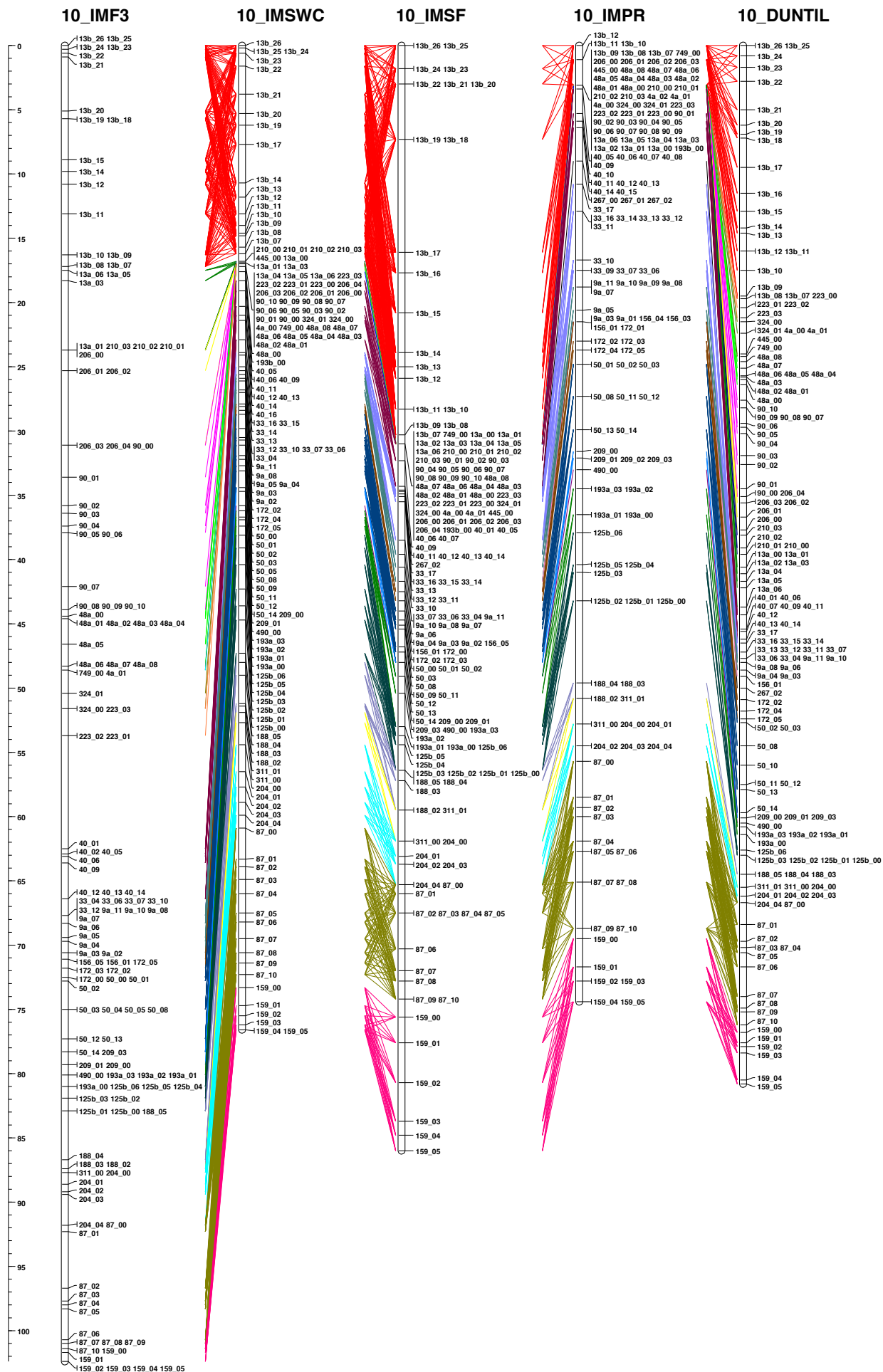

Supplement: S11 Fig — Each genomic scaffold is given in a separate color. (PDF) [file pcbi.1006949.s012.pdf]

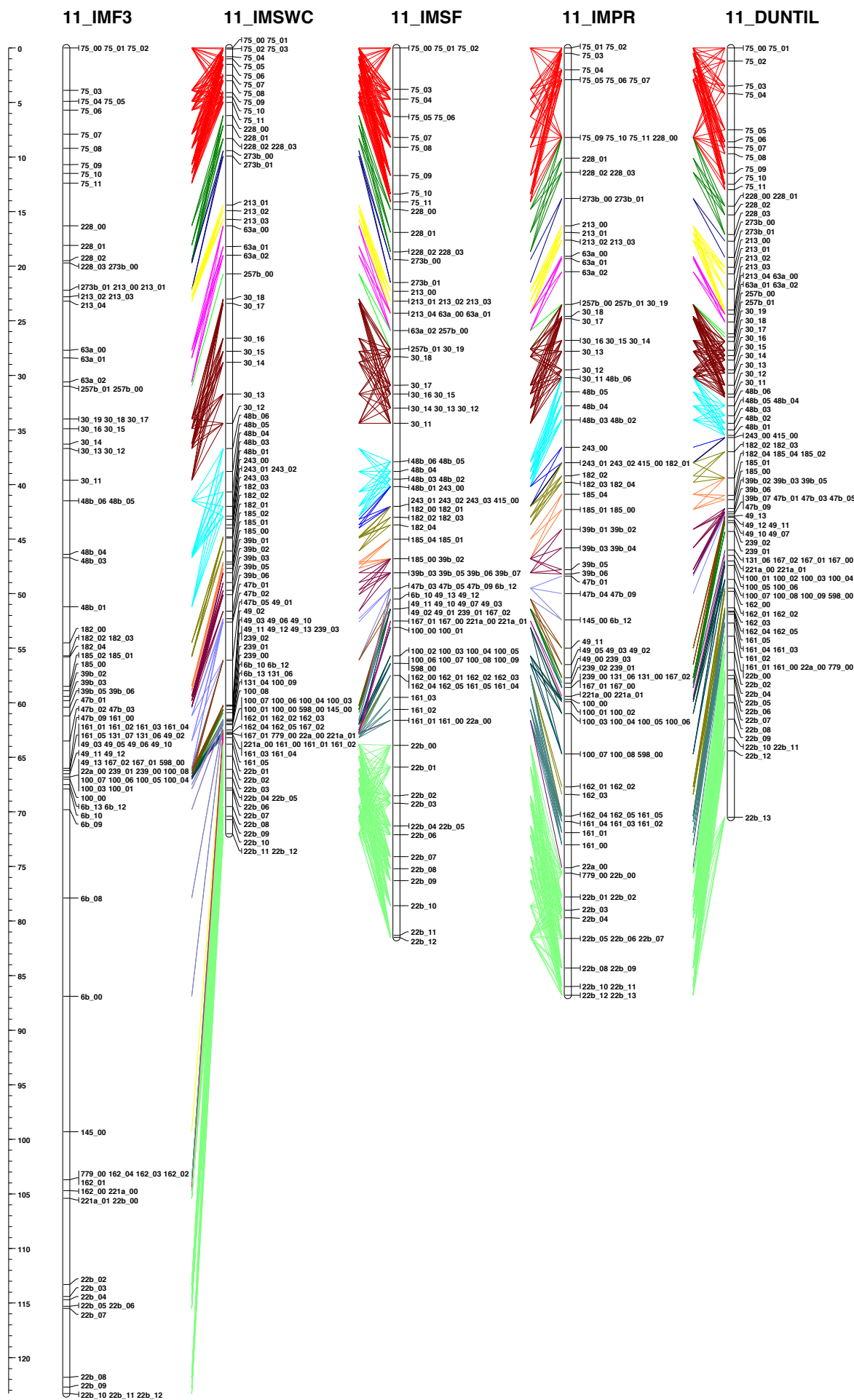

Supplement: S12 Fig — Each genomic scaffold is given in a separate color. (PDF) [file pcbi.1006949.s013.pdf]

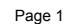

Supplement: S13 Fig — Each genomic scaffold is given in a separate color. (PDF) [file pcbi.1006949.s014.pdf]

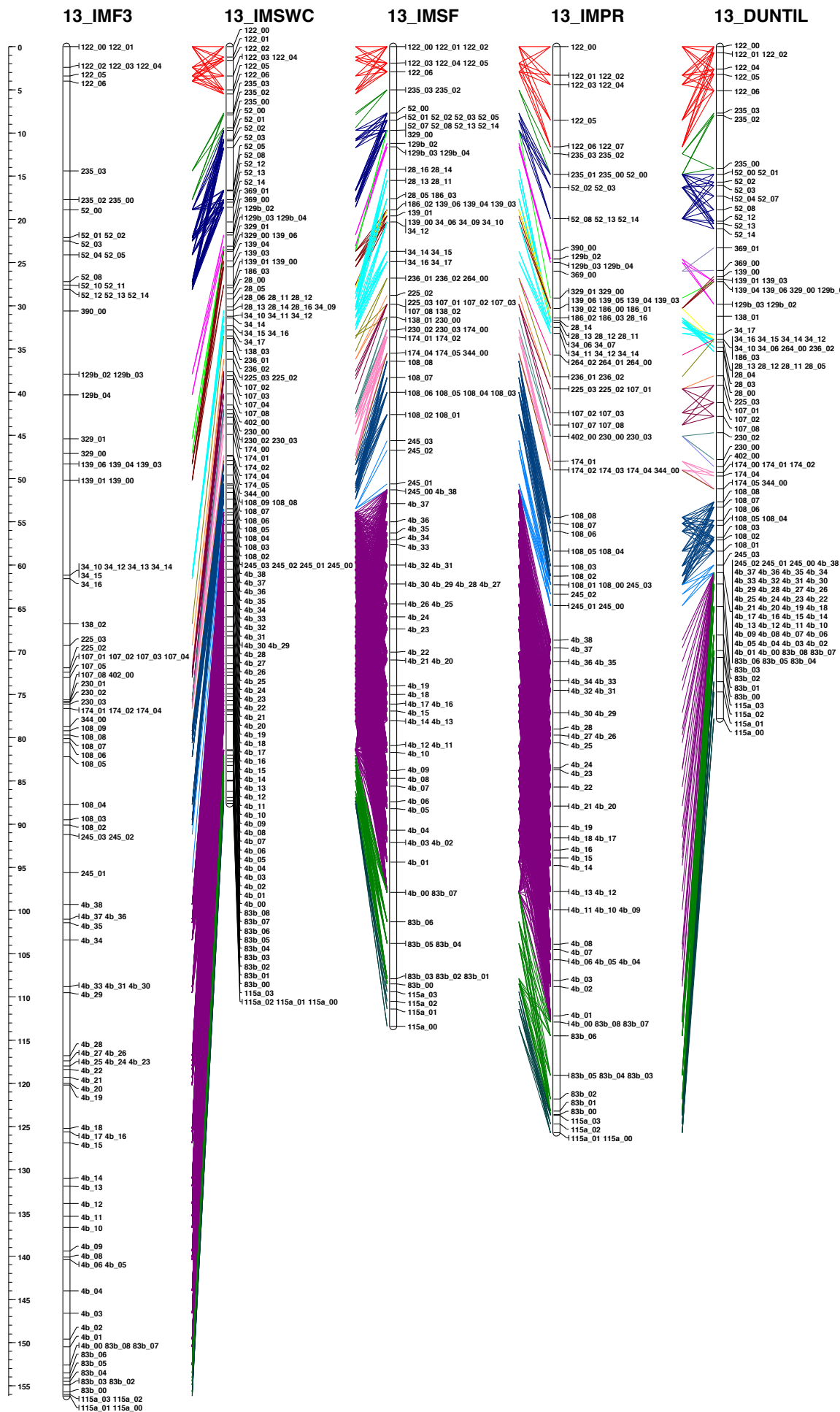

Supplement: S14 Fig — Each genomic scaffold is given in a separate color. (PDF) [file pcbi.1006949.s015.pdf]

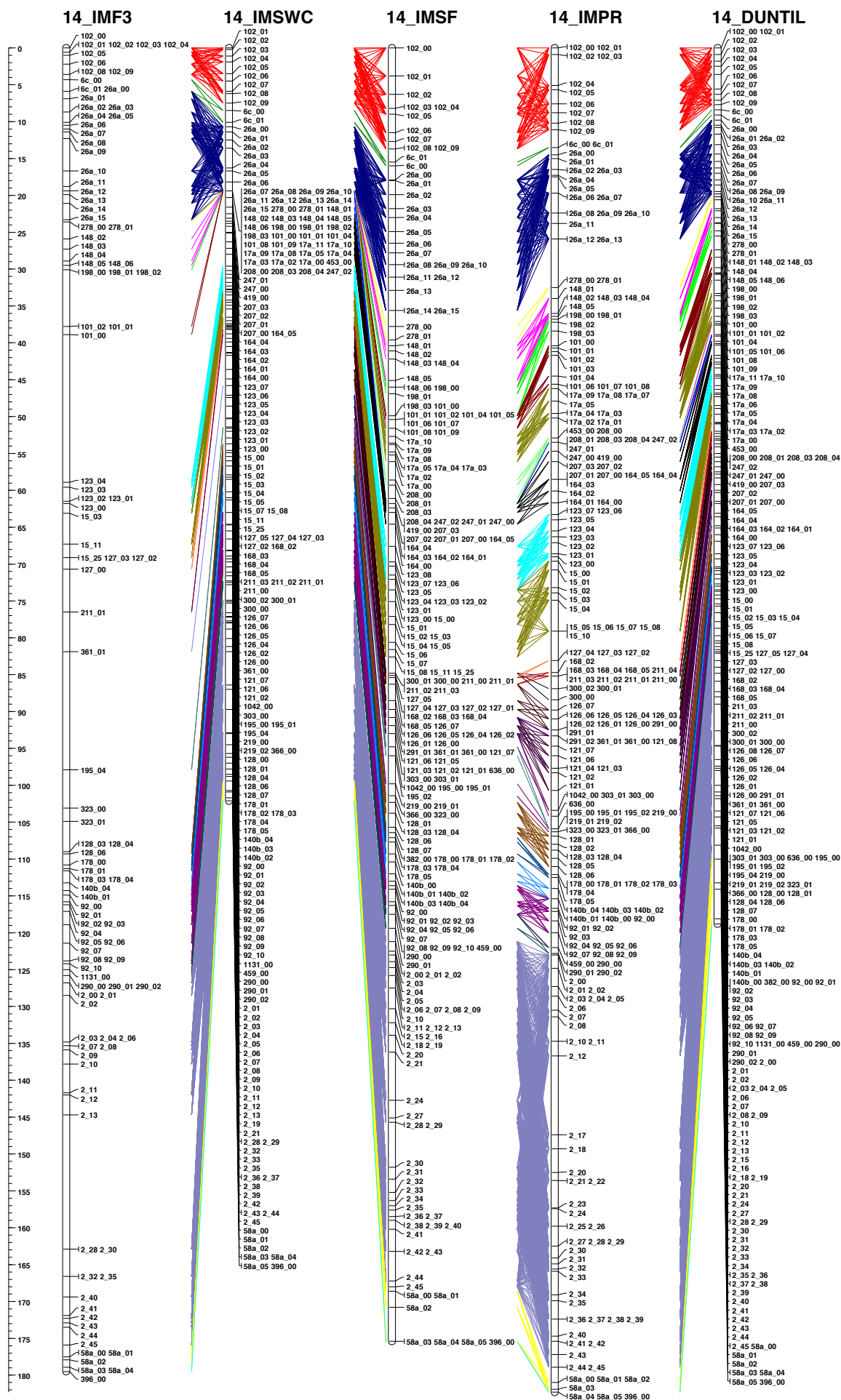

Supplement: S15 Fig — Each genomic scaffold is given in a separate color. (PDF) [file pcbi.1006949.s016.pdf]
