# Supplementary material for: GOOGA: A platform to synthesize mapping experiments and identify genomic structural diversity
Source: PLoS Comput Biol. 2019 Apr 15;15(4):e1006949. doi: 10.1371/journal.pcbi.1006949 (PMC6483263; doi:10.1371/journal.pcbi.1006949)
Supplement: S16 Fig — Chromosome diagrams for all five crosses are listed each in a separate panel, with each parent listed to the right of the diagram. The inversion karyotype is indicated by color (red or blue) and by either ‘>>>>>>>‘ or ‘<<<<<<<‘. The IMF3 and DUNTIL crosses were both formed by homokaryotypic parents and show free recombination. Thus these parents can be inferred as karyotypes A (IMF3) or B (DUNTIL). The next three crosses represent A/B heterokaryotypes and show strong recombination suppression. However, because parents from the IMF3 cross (A karyotype) were used in these crosses, we can infer that these parents are A and that the other must be B. (PDF) [file pcbi.1006949.s017.pdf]

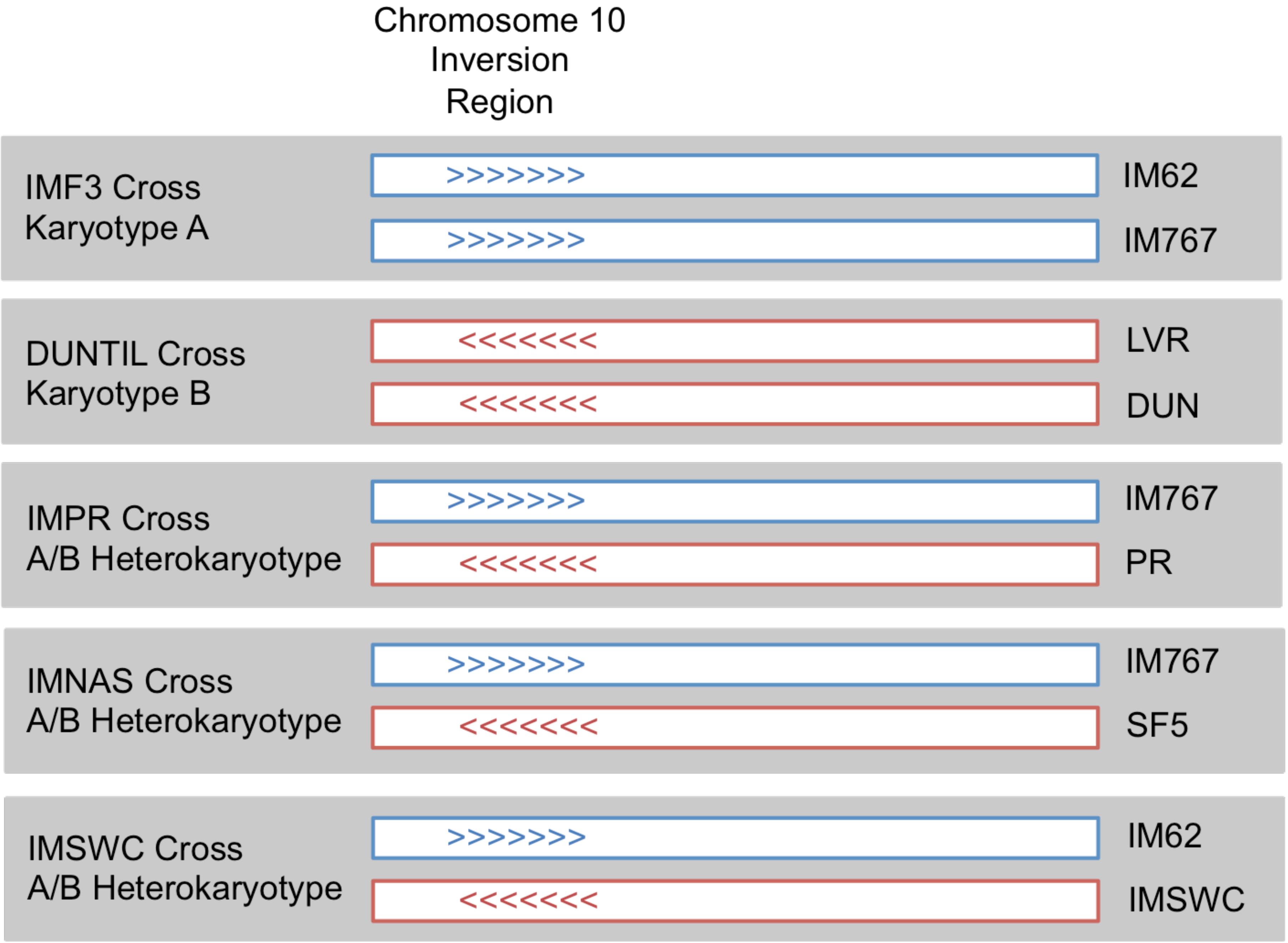
Chromosome 10
Inversion
Region
IMF3 Cross
Karyotype A
IM62
IM767
DUNTIL Cross
Karyotype B
LVR
DUN
IMPR Cross
A/B Heterokaryotype
IM767
PR
IMNAS Cross
A/B Heterokaryotype
IM767
SF5
IMSWC Cross
A/B Heterokaryotype
IM62
IMSWC
