# Supplementary material for: GOOGA: A platform to synthesize mapping experiments and identify genomic structural diversity
Source: PLoS Comput Biol. 2019 Apr 15;15(4):e1006949. doi: 10.1371/journal.pcbi.1006949 (PMC6483263; doi:10.1371/journal.pcbi.1006949)
Supplement: S2 Table — (DOCX) [file pcbi.1006949.s019.docx]

Supporting Table 2

| Species (ecotype) | Population | Lines | proposed inversions |
| --- | --- | --- | --- |
| *M. guttatus (annual)* | Iron Mountain (IM) |  | LG10 vs. non-IM |
|  |  | IM62 ref | LG11 vs. all others |
|  |  | IM767 |  |
| *M. guttatus (annual)* | Sweet Creek (SWC) | SWC |  |
| *M. nasutus (annual)* | Sherars Falls (SF) | SF5 |  |
| *M. guttatus (perennial)* | Florence Dunes (DUN) | DUN10 | LG8, LG5 vs. annuals/LVR |
| *M. guttatus (perennial)* | Point Reyes (PR) | PR | LG8, LG5 vs. annuals/LVR |
| *M. tilingii* | LVR | LVR | LG13 vs. DUN |
