# Supplementary material for: GOOGA: A platform to synthesize mapping experiments and identify genomic structural diversity
Source: PLoS Comput Biol. 2019 Apr 15;15(4):e1006949. doi: 10.1371/journal.pcbi.1006949 (PMC6483263; doi:10.1371/journal.pcbi.1006949)
Supplement: S1 Appendix — (DOCX) [file pcbi.1006949.s024.docx]

***Supplemental Appendix 1***

**Generating putative genotype calls in five Mimulus experiments**

***Genomic scaffolds of M. guttatus—***The first iteration of the *M. guttatus* reference genome (v1, available at https://phytozome.jgi.doe.gov; login required) was obtained from sequencing of a single inbred line (IM62; Iron Mountain, OR, USA) and consists of over 2000 scaffolds. The longest are greater than 4 Mb in length (about 15% of an average *M. guttatus* chromosome) but the majority of sequence is contained in scaffolds 10 kb - 1 Mb in size. The current assembly (V2 reference genome: available at https://phytozome.jgi.doe.gov; login required) orients most of the v1 scaffolds into chromosomal groups based on multiple data sources [1]. For the present analysis, we revert back to the v1 scaffolds as a target for mapping sequence reads from recombinant individuals. We retain the set of breaks that were made to v1 scaffolds when creating the V2 build given that these breaks were largely corroborated by a subsequent mapping study [2]. We append a letter to names for broken v1 scaffolds, e.g. scaffold_97 is now scaffold_97a, scaffold_97b, and scaffold_97c. Next, we appended the mitochondrial and chloroplast genomic contigs to the scaffold list and masked repetitive regions to produce our read mapping target: Updated_v1_hardmasked.fa. The autosomal v1 scaffolds are the basic units for subsequent work. Markers are defined as contiguous stretches of DNA within these scaffolds.

***Creation and Genotyping of Mapping populations—***Five crosses are analyzed in this study (Table 1). Three crosses are F2s (IMNAS, IMSWC, DUNTIL), one is an F3 (IMF3), and one is a Recombinant Inbred Line panel (IMPR). The IMF3 population was founded by crossing two highly homozygous lines (IM62 and IM767) sampled from one population (Iron Mountain, OR, USA). A single F1 plant was selfed to create many F2s. F2 plants were randomly paired and crossed to produce F3 seed. Over 1000 F3 hybrids were grown to maturity and a random subset of these plants were genotyped for map construction. The IMSWC cross was formed from a cross between IM62 and SWC (Table 1), an annual plant from a population of *M. guttatus* from Mapleton, OR, USA. The DUNTIL cross was formed by crossing an inbred line of *M. guttatus* (DUN10; Florence, OR, USA) to an inbred line of *Mimulus tilingii* (LVR; Inyo, CO, USA) [3]. For both IMSWC and DUNTIL, a single F1 plant was self-fertilized to create the F2 plants that were subsequently genotyped. IMNAS is a tri-parental cross: Two inbred lines from Iron Mountain (OR, USA) *M. guttatus* were each crossed to the SF5 (Sherars Falls, OR, USA) inbred line from selfing species *Mimulus nasutus*. The two interspecific F1s were then intercrossed [(SF5xIM160) x (SF5xIM767)] to produce the F2 plants. Because IM160 (like the reference line IM62) carries the driving *D* centromeric variant of LG11, which transmits nearly 100% via female function in heterozygotes with the *M. nasutus* *d* variant [4], this F2 segregates as a backcross in the D region. Finally, the IMPR are a set of highly homozygous lines derived from serially selfing progeny from a cross between IM767 and PR from the Point Reyes, CA, population of *M. guttatus* (see [5] for a description of RIL line formation).

***Library preparation, Genotyping, and SNP identification—***The DNA extraction method and procedures for genotyping individuals using the Multiplexed-Shotgun-Genotyping (MSG; [6]) are described in Holeski et al [2]. Briefly, we digested DNA from each sample using a restriction enzyme (MseI or AseI) and then ligated unique Bar-Coded-Adapters (BCAs) to the resultant DNA fragments. After numerous cleaning steps, we size-selected libraries for fragments between 250-425 bp. We then performed PCR reactions (14-18 cycles) using Phusion High-Fidelity PCR Master Mix and primers that bind to common regions in the BCAs. This elongates the molecules to contain necessary flanking sequence including the Illumina adaptors as well as additional indices to allow further multiplexing of samples within a single sequencing lane. Subsequent sequencing was performed using the Illumina instrument. MseI was used for the restriction digest to make a library for each the five mapping populations. We made a second library for the IMF3 population with a less frequent cutter, AseI. The genotype calls for IMF3 are synthesized from sequencing on these two libraries (see below). Details regarding the sequencing for DUNTIL [3], IMPR [2], IMNAS (Finseth et al, 2018, in prep) and IMSWC (Kooyers et al, 2018, in prep) are reported elsewhere, though briefly DUNTIL and IMSWC were sequenced at the Duke University Center for Genomic and Computational Biology, IMPR and IMF3 were sequenced at the University of Kansas Genome Sequencing Core, and IMNAS was sequenced at Hudson Alpha Genomic Services Laboratory.

Following sequencing, we demultiplexed reads into sample specific fastq files and remove adaptor contamination with Scythe (<https://github.com/vsbuffalo/scythe/>) and trimmed low quality sequence with Sickle (<https://github.com/najoshi/sickle/>). We used the *mem* function of BWA [7] to map read (or read pairs), one sample at a time, to Updated_v1_hardmasked.fa. Following read mapping, we identified putative SNPs using the UnifiedGenotyper function of the Genome Analysis ToolKit (GATK; [8]).

For each mapping population, we started with a vcf file with all recombinant individuals scored for SNPs within individual reads or read pairs (RADtags) and whole-genome sequencing data from one or both parental lines. We eliminated SNPs with a mapping quality score less than 30 or a minor allele frequency less than 0.1. We further thinned the data to a single SNP per RADtag, selecting the one with the most scored individuals. After inspecting the distribution of reads called to each parent per individual for each possible read depth, we set a maximum median depth (across individuals scored for a SNP) specific to each mapping population: 10 for IMPR, 3 for IMSWC, 4 for DUNTIL, 10 for IMNAS, 5 for MseI libraries of IMF3, 20 for AseI libraries of IMF3. SNPs with excessive depth were suppressed.

***Assigning parent of origin to SNPs—***Denoting the two parents of a mapping population as A or B, alleles were scored by assigning the reference base at each SNP to a specific parent (A or B). This was possible because one or both the parents in each crosses was MSG genotyped and/or fully genome sequenced. For the IMF3 population, one parent (IM62) is the reference genome sequence and the other (IM767) has been fully sequenced [9]. IM62 was also a parent in the IMSWC cross and the alternative base at a SNP is necessarily from the second parent (SWC). We used the IM767 genome sequence to polarize bases in the IMPR. For DUNTIL, we used genome sequences from both parents (LVR and DUN10) to specify base ancestry in the F2s [3]. For the IMNAS cross, we limited consideration to SNPs where an F1 hybrid between the IM767 and IM160 parents was homozygous for one base and the SF5 parent was homozygous for the alternative. After establishing the parent for each SNP along each v1 scaffold, we imposed another layer of filtering based on consistency of the inferred parent within a recombinant individual along v1 scaffolds. We expect the same parentage of closely linked SNPs: If an individual is AA at a SNP, it should usually be AA at neighboring SNPs (excepting occasional recombination). SNPs exhibiting excessive disagreement were eliminated (details below).

Window based genotyping calling was then performed as described in main paper. The resulting genotype file for each individual is AA/AB/BB/NN at each marker. We imposed a third layer quality filtering at the scale of window-based genotypes. Each marker was scored for genotype frequencies and agreement of markers within 1 Mb windows. We eliminated markers that were excessively heterozygous across mapping populations and/or exhibited high disagreement with neighboring markers. Within each mapping population, we suppressed loci that were excessively heterozygous in that population and/or had low numbers of called individuals. Finally, we imposed a cross-specific minimum number of called loci for a plant to be included in subsequent mapping.

***Initial maps—***The initial maps input to GOOGA were based on the V2 build. We tested the assignment of scaffolds as predicted by the V2 build and found them consistent with our genotyping data (markers on the same linkage group exhibit positive association of genotypes). However, we were also able to tie an additional 26 v1 scaffolds to linkage groups which were unassigned in the V2 build (Supporting Table 6).

1. Hellsten U, Wright KM, Jenkins J, Shu S, Yuan Y, Wessler SR, et al. Fine-scale variation in meiotic recombination in Mimulus inferred from population shotgun sequencing. Proc Natl Acad Sci. 2013;110. doi: 10.1073/pnas.1319032110.

2. Holeski L, Monnahan P, Koseva B, McCool N, Lindroth RL, Kelly JK. A High-Resolution Genetic Map of Yellow Monkeyflower Identifies Chemical Defense QTLs and Recombination Rate Variation. G3: Genes|Genomes|Genetics. 2014;4(5):813-21. doi: 10.1534/g3.113.010124.

3. Garner AG, Kenney AM, Fishman L, Sweigart AL. Genetic loci with parent-of-origin effects cause hybrid seed lethality in crosses between Mimulus species. New Phytologist. 2016;211(1):319-31. doi: 10.1111/nph.13897.

4. Fishman L, Saunders A. Centromere-Associated Female Meiotic Drive Entails Male Fitness Costs in Monkeyflowers. Science. 2008;322(5907):1559-62.

5. Holeski LM, Chase-Alone R, Kelly JK. The Genetics of Phenotypic Plasticity in Plant Defense: Trichome Production in Mimulus guttatus. American Naturalist. 2010;175(4):391-400. doi: 10.1086/651300. PubMed PMID: ISI:000275167100001.

6. Andolfatto P, Davison D, Erezyilmaz D, Hu TT, Mast J, Sunayama-Morita T, et al. Multiplexed shotgun genotyping for rapid and efficient genetic mapping. Genome research. 2011;21(4):610-7. doi: 10.1101/gr.115402.110. PubMed PMID: WOS:000289067800011.

7. Li H, Durbin R. Fast and accurate short read alignment with Burrows-Wheeler Transform. Bioinformatics. 2009;25:1754-60.

8. McKenna A, Hanna M, Banks E, Sivachenko A, Cibulskis K, Kernytsky A, et al. The Genome Analysis Toolkit: A MapReduce framework for analyzing next-generation DNA sequencing data. Genome Research. 2010;20(9):1297-303. doi: 10.1101/gr.107524.110.

9. Flagel LE, Willis JH, Vision TJ. The standing pool of genomic structural variation in a natural population of Mimulus guttatus. Genome biology and evolution. 2014;6(1):53-64.
