# Supplementary material for: GOOGA: A platform to synthesize mapping experiments and identify genomic structural diversity
Source: PLoS Comput Biol. 2019 Apr 15;15(4):e1006949. doi: 10.1371/journal.pcbi.1006949 (PMC6483263; doi:10.1371/journal.pcbi.1006949)
Supplement: S2 Appendix — (DOCX) [file pcbi.1006949.s025.docx]

***Supplemental Appendix 2***

**Model details**

***Transition probabilities for each experimental design—***In an F_2_ population, the probabilities are (1-r)^2^, 2r(1-r), and r^2^ for AA transitioning to AA, AB, and BB, respectively [1]. Here, r is the recombination rate and is specific to the flanking markers. The transition probability of AB to AB is (1-r)^2^ + r^2^ and the probability AB to either homozygote is 2r(1-r). The model is symmetric: The probabilities are (1-r)^2^, 2r(1-r), and r^2^ for BB transitioning to BB, AB, and AA, respectively. An additional round of recombination occurs in an F_3_ population in gamete formation by F_2_s. However, the probabilities have the same form except with an expected 50% increase in recombination rate values (assuming no change in crossover rates between F1s and F2s). In the RILs, heterozygosity has largely been eliminated by inbreeding. For analysis of the Mimulus data, we suppress the remaining heterozygous regions by calling NN at those loci. For the resultant genotypes, we stipulate the transitions probabilities as (1-r) and r for AA transitioning to AA and BB, respectively. For both RILs and F3s, the r parameter is actually a composite from multiple meioses. These apparent recombination rates are distinct from r of the F_2_ populations (the expected proportion of recombinant gametes from one round of meiosis) [2]. Thus, while the same markers are present across the different types of mapping populations, the absolute value of r will vary according to cross type.

***Defining mapping populations following genotyping error estimates—***We used error rate estimates to cull plants with highly flawed genotypes from subsequent analyses. For example, there were 130 F2s in the IMNAS population that passed preceding filters for genotyping error rates. After excluding all plants where (e0i + e1i + e2i) ≥ 0.1, we obtained the set of 91 plants used for all downstream analyses. The post-error rates estimation sample sizes are 181 for IMF3, 872 for IMSWC, 205 for DUNTIL, and 260 for IMPR.

1. Fisher R, Balmukand B. The estimation of linkage from the offspring of selfed heterozygotes. Journal of Genetics. 1928;20(1):79–92. doi: doi:10.1007/BF02983317.

2. Martin OC, Hospital F. Two- and Three-Locus Tests for Linkage Analysis Using Recombinant Inbred Lines. Genetics. 2006;173(1):451-9. doi: 10.1534/genetics.105.047175.
