# Supplementary material for: GOOGA: A platform to synthesize mapping experiments and identify genomic structural diversity
Source: PLoS Comput Biol. 2019 Apr 15;15(4):e1006949. doi: 10.1371/journal.pcbi.1006949 (PMC6483263; doi:10.1371/journal.pcbi.1006949)
Supplement: S3 Appendix — (DOCX) [file pcbi.1006949.s026.docx]

***Supplemental Appendix 3***

**Genetic algorithm implementation**

***Mating, mutation, and recombination—***After calculating fitnesses, all *N* individuals were sorted in ascending order by likelihood and each individual (*i*) was assigned a rank (*R_i_*) of 1 to *N*.  The probability of selecting an individual on the basis of its rank was $P\left( i \right)={R_{i}}/{\sum_{k=1}^{N} R_{k}}$. Pairs of unique individuals were randomly selected based on these probabilities, and subjected to mutation and recombination operations. We used a mutation scheme called “swap mutation”, where one individual was randomly selected from the pair, and then the location of up to four pairs of scaffolds within that individual were randomly swapped (while also flipping the orientation 50% of the time), thus creating a new “mutant” scaffold order. The mutated individual was then recombined with the other member of the pair.  Recombination was performed using a scheme called the “order crossover”.  This involves first choosing a random length segment of scaffolds from the donor individual and randomly inserting it into the scaffold order of the recipient individual to make a new individual.  Each scaffold contributed by the donor is now duplicated in the recipient individual.  To fix this we deleted all the recipient individual’s copies of the duplicated scaffolds, while preserving the relative order of the non-duplicates. The swap mutation and ordered crossover do not mimic the mechanics of biological mutation or meiotic recombination. However both create diverse new individuals (i.e. scaffold orders) while preserving partial solutions from both the donor and recipient individuals. This allows the GA to build on past successes while exploring new scaffold orders.

***Computation—***The calculations were parallelized so that the HMM could be run simultaneously on each individual in the population.  To further speed up the HMM, we implemented a memoization strategy that stored past recombination fraction (RF) results between scaffolds and injected those precomputed results into the future HMM calculations to avoid recalculation. To understand the mechanism of the memoization strategy, as a toy example and for the moment ignoring scaffold orientation, imagine a chromosome with four scaffolds A, B, C, and D and an initial individual ordered ABCD with recombination rates computed between A-B, B-C, and C-D. After calculating the recombination rates for this individual, we store the rate for the entire length (ABCD) and the rates of all suborders of at least two scaffolds (ABC, BCD, AB, BC, CD), to create a catalog of previously computed rates. Then, when a future individual is produced, say DABC, we would search the catalog for matches starting with the longest members (ABCD) and then the next longest (ABC, BCD), and so on until the catalog is exhausted. For DABC, the first match would be the suborder ABC, which would supply recombination rates for A-B and B-C, leaving D-A as the only missing rate to be calculated. After HMM calculation, all new suborders (DABC, DAB, and DA) would be added to the catalog for future use. This approach greatly speeds up calculation, especially at the later stages of optimization when many individuals tend to have large tracts of identical scaffold orders. We found that this approach reliably underestimates the likelihood value by a small amount when compared to the slower processes of calculating all recombination rates *de novo* for every new individual (among 750 random test samples all memoization estimates had a lower lnLK, with a median underestimate of 0.02%). This means the memoization method is consistently slightly conservative. For this reason, when a new individual was found to be among the elites (i.e. a promising new scaffold order), the program paused and recomputed this individual’s exact likelihood without precomputed recombination rates and used this more precise likelihood for future ranking. The GA procedure is implemented in Python (version 2.7), utilizing functions from the scipy library (http://www.scipy.org/). The code is open source and available online ([https://github.com/flag0010/GOOGA](https://github.com/flag0010/GenomeMapping)).  For the Mimulus experiments, all GA runs were initiated with a map based on the V2 genome order. For the Drosophila simulation, we created the initial maps using make.ends.meet.py. A GA run was configured to terminate after 1000 generations with no change to the highest likelihood map or after running for 96 hrs.
